# Metabolic and immunological responses of *Drosophila melanogaster* to dietary restriction and bacterial infection differ substantially between genotypes in a population

**DOI:** 10.1002/ece3.8960

**Published:** 2022-05-24

**Authors:** Wesam S. Meshrif, Sandy H. Elkayal, Mohamed A. Soliman, Amal I. Seif, Thomas Roeder

**Affiliations:** ^1^ Department of Zoology Faculty of Science Tanta University Tanta Egypt; ^2^ Faculty of Pharmacy Pharmaceutical Services Center Tanta University Tanta Egypt; ^3^ Department of Molecular Physiology Zoological Institute Kiel University Kiel Germany; ^4^ Airway Research Center North German Center for Lung Research Kiel Germany

**Keywords:** diversity, fruit fly, infection, restricted diet, trait plasticity

## Abstract

To respond to changing environmental conditions, a population may either shift toward better‐adapted genotypes or adapt on an individual level. The present work aimed to quantify the relevance of these two processes by comparing the responses of defined *Drosophila melanogaster* populations to different stressors. To do this, we infected two homogeneous populations (isofemale lines), which differ significantly in fitness, and a synthetic heterogeneous population with a specific pathogen and/or exposed them to food restriction. *Pectobacterium carotovorum* was used to infect *Drosophila* larvae either fed standard or protein‐restricted diet. In particular, the two homogeneous groups, which diverged in their fitness, showed considerable differences in all parameters assessed (survivorship, protein and lipid contents, phenol‐oxidase (PO) activity, and antibacterial rate). Under fully nutritious conditions, larvae of the homogeneous population with low fitness exhibited lower survivorship and protein levels, as well as higher PO activity and antibacterial rate compared with the fitter population. A protein‐restricted diet and bacterial infection provoked a decrease in survivorship, and antibacterial rate in most populations. Bacterial infection elicited an opposite response in protein and lipid content in both isofemale lines tested. Interestingly, the heterogeneous population showed a complex response pattern. The response of the heterogeneous population followed the fit genotype in terms of survival and antibacterial activity but followed the unfit genotype in terms of PO activity. In conclusion, our results show that defined genotypes exhibit highly divergent responses to varying stressors that are difficult to predict. Furthermore, the responses of heterogeneous populations do not follow a fixed pattern showing a very high degree of plasticity and differences between different genotypes.

## Graphical abstract

The response of two genotypes was compared with a heterogeneous population of *Drosophila melanogaster*. The populations were raised under four environments regarding diet restriction and bacterial infection. Plasticity may enable the genotypes to withstand the environmental changes before exhibiting a genetic change upon adaptation.

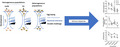

## 1 INTRODUCTION

One important mechanism by which animals can respond to adverse conditions, like scarce nutrition, exposure to pathogens or parasites, or global warming, is the phenotypic plasticity of a given genotype (Flatt, 2020). Phenotypic plasticity can manifest as differences in life history, behavior, or physiology when individuals are exposed to different environmental conditions (Schlichting & Pigliucci, 1998). This plasticity is essential for enhancing the survival of genotypes or populations exposed to environmental stress (Whitman & Agrawal, 2009). Knowledge of the plasticity of animal and plant response patterns is of considerable importance for predicting their responses to environmental change and community dynamics (Oms et al., 2017; Valladares et al., 2006). On the other hand, canalization is a process whereby individuals may express phenotypic consistency in face of environmental and/or genetic perturbation (Debat & Le Rouzic, 2019).

Differences in population responses could be due to intrinsic factors, such as genetic differences among the genotypes that make up that population or correspond to differential response patterns of specific genotypes within a population. A very good example of this type of reaction is provided by host–parasite interactions, where parasites usually tend to increase their virulence in successive generations, while hosts increase their resistance (Vrijenhoek, 1986). Here, the host diversity (homogeneous vs. heterogeneous) may decrease or increase the disease risk within and among populations (Zargar et al., 2015). In addition to genetic variation, extrinsic factors such as nutrition greatly influence the resistance of hosts to pathogens (Boeing, 2013). Most relevant among these extrinsic factors are nutritional factors such as malnutrition for which increased susceptibility to infectious diseases was documented, as nutrient deficiency impairs the host's immune response (Ponton et al., 2020). Consequently, immune defense against infection is thought to be an important component of fitness in most organisms and can be strongly impacted by environmental conditions (Hawley & Altizer, 2011).

*Drosophila melanogaster* is considered a prime model to study the differential effects of phenotypic variation in response to environmental changes (Elkayal et al., 2016; Meshrif & Elkholy, 2015; Schneider, 2000). It was shown that the nutritional quality of the diet during development influences the ability of *Drosophila* to either resist or tolerate pathogens (Cotter et al., 2019; Howick & Lazzaro, 2014). The diet of *Drosophila* mainly consists of sugar and yeast as a source of proteins and amino acids. Several studies elucidated the effect of a diet dilution or imbalanced diets on the flies’ longevity, fecundity, mortality, and immune reactions (Ellers et al., 2011; Howick & Lazzaro, 2014; Min et al., 2007). It appears that yeast takes an important role in nutritional content ensuring proper development and survival (Elkayal et al., 2016). Surprisingly, our knowledge about the effect of multiple environmental stressors like the combination of infection and different nutritional stressors in the context of different genotypes is to be assessed as being very low. To resist environmental stressors like diet restriction or bacterial infection, the organism uses the energy available (Rion & Kawecki, 2007). Thus, allocating energy is vital to mounting an effective immune reaction which is costly and might interfere with other relevant traits such as the metabolic reserves (Ellers et al., 2011). Body carbohydrates, lipids, and proteins may be essential as a source of energy for immune reactions and help to extend longevity and stress resistance (Djawdan et al., 1998; Mullen & Goldsworthy, 2003; Thompson, 2003 **)**.

In the current study, we utilized a straightforward design to elucidate the differential contribution of genotypes in response to diet restriction or bacterial infection and their combinations. Therefore, we used two different homogeneous (full‐sib mating) lines that differ substantially in a major life‐history trait and compared them with a heterogeneous (synthetic) population that reflects the genetic diversity in the original population. These different populations were subjected to immune and nutritional stressors and the combination of both and their reactions toward these stressors was quantified using comprehensive phenotyping. *Pectobacterium carotovorum* (formerly *Erwinia carotovora*), the bacterial pathogen is a phytopathogen (Agrios, 1997; Basset et al., 2000), is proven to infect *D*. *melanogaster* during feeding and cause chronic infection in the gut (Vieira, 2014). Due to its persistence in the gut, it can trigger a local and systemic immune response (Basset et al., 2000; Lemaitre & Hoffmann, 2007).

## 2 MATERIALS AND METHODS

### 2.1 Fly strains and fly husbandry

**FIGURE 1:**
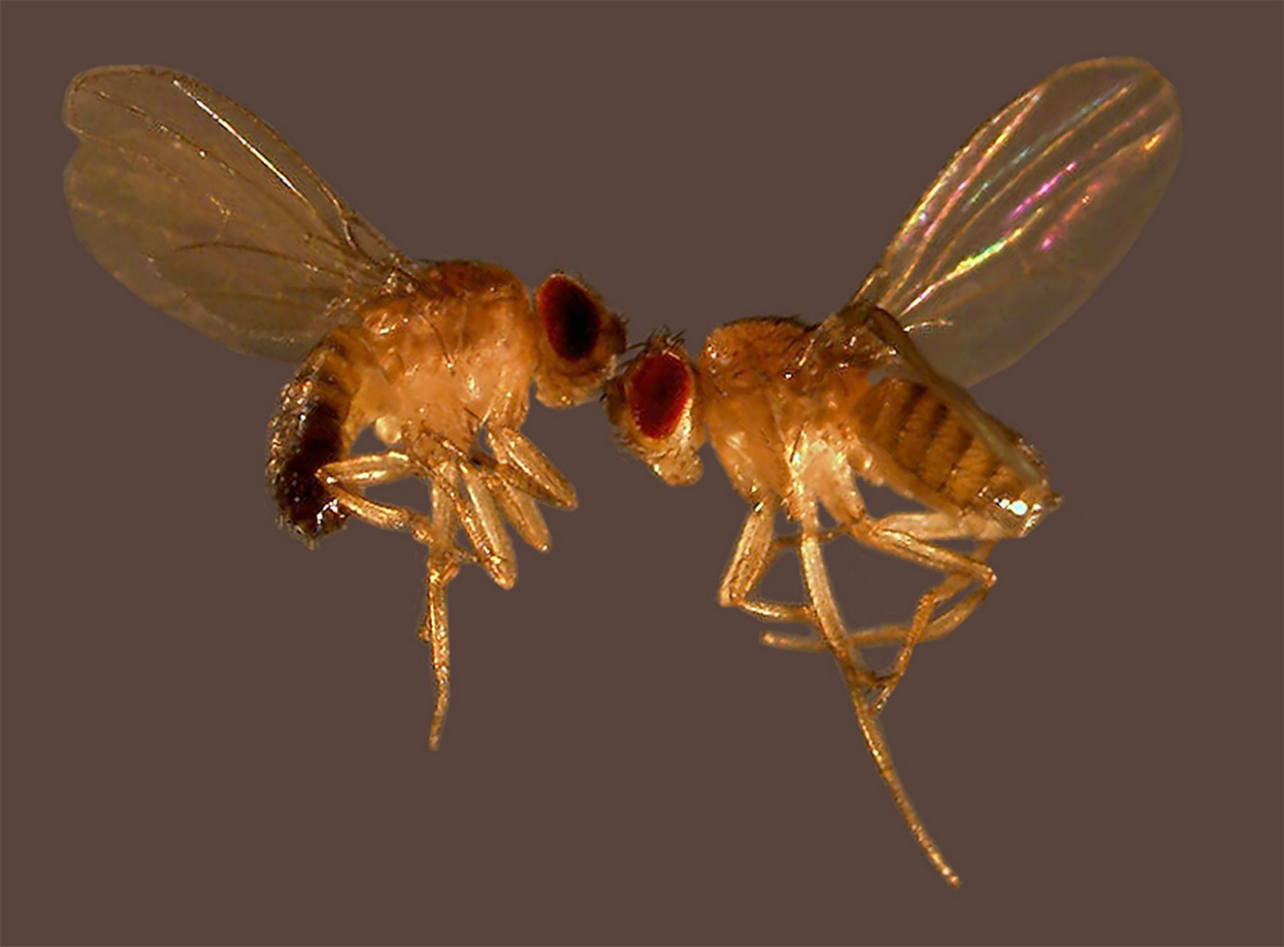
Photomicrograph of adult male and female of wildtype *Drosophila melanogaster* originated in Zimbabwe

**FIGURE 2:**
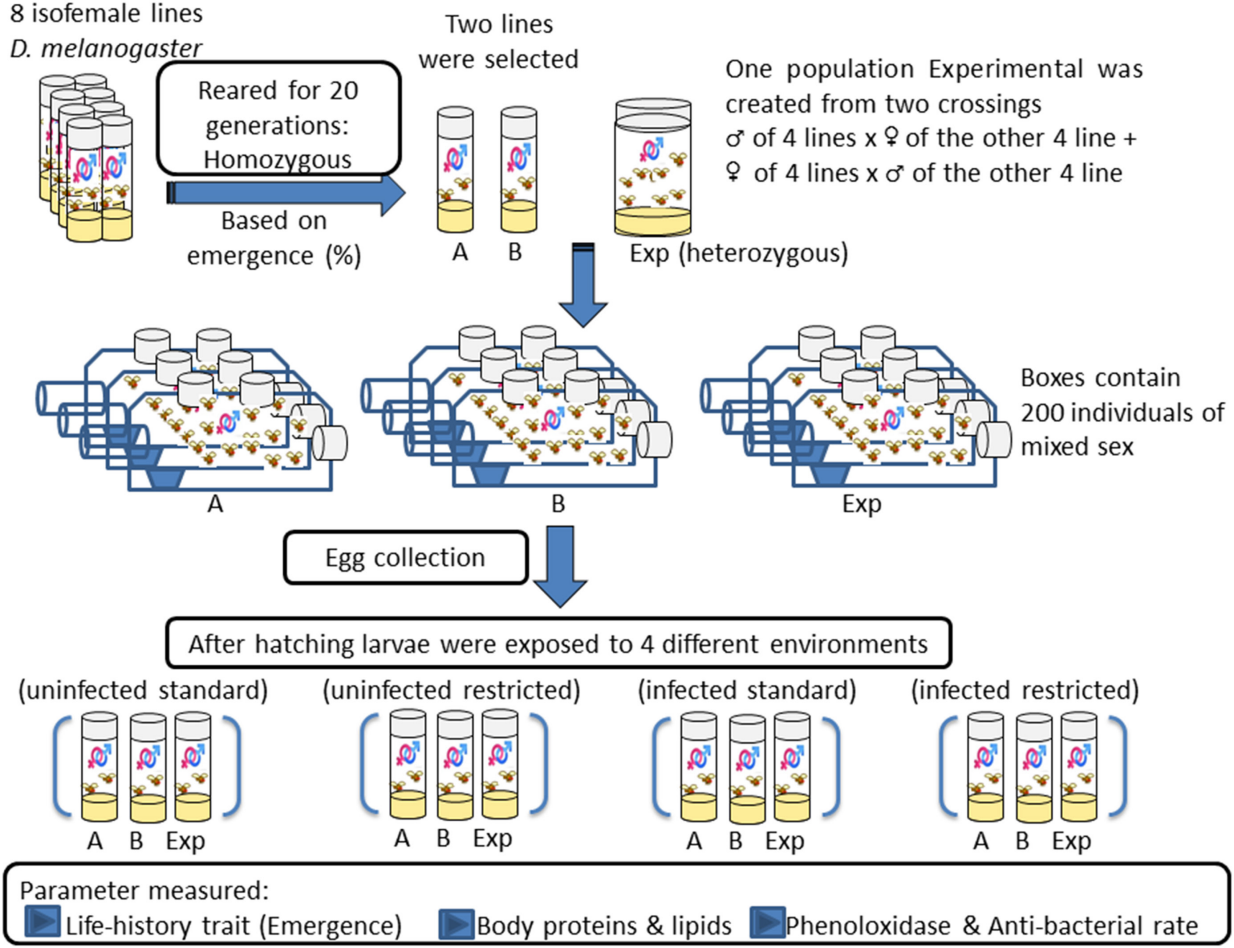
Diagram summarized the experimental design in the study. 8 *Drosophila melanogaster* isofemale lines were reared on sugar‐cornfloor‐yeast media in standard vials for 20 generations to have homogenous populations. Thereafter one experimental population was created from these 8 lines by making two crossings: one between the males of 4 lines and the females of the other 4 lines and another crossing between the female of the first 4 lines and the males of the other 4 lines. The offspring of both crossings were mixed to have all traits in these lines (heterogeneous population). In addition to the heterogeneous population, two extreme lines regarding survivorship denoted A and B were used to measure the variation of their response under different environmental conditions. A3–5 cages of all the 3 populations were created; every cage contained 200 individuals mixed‐sex. After egg collection from these cages using hard‐agar medium, the 1st instar larvae of these populations were let to develop in the different environments regarding aseptic condition and diet: aseptic condition with a standard diet, aseptic condition with a restricted diet, infected condition with a standard diet, and infected condition with a restricted diet

Eight (inbred) isofemale lines of African *D*. *melanogaster* (origin: Zimbabwe) were used as different genotypes and represented homogeneous populations (Figure 1). These populations were full‐sib matting for 17 generations to reach homozygosity 97% (Ashburner, 1989). These stock lines were maintained for a minimum of 20 generations in vials containing 50 individuals of mixed sex. A total of 200 individuals (mixed sex) of each line were diffused together in a plastic cage (28 × 20 × 14 cm) to prevent genetic drift. Flies were reared on a standard medium consisting of 63 g/L each of cornmeal, sucrose, and yeast in addition to 12.5 g/L of agar (Meshrif & Elkholy, 2015). Stock lines were maintained at 25 ± 2°C, 60%–80% RH, under a 12 h light:12 h dark cycle and aseptic conditions. Two isofemale lines were selected for use in the present study based on emergence percentage (survivorship) and referred to as A and B. Line No 186 was denoted as population A that produced the lowest egg to adult survival rate while Line No 229 was denoted as population B that produced the highest egg to the adult survival rate of the eight isofemale lines tested as reported in Elkayal et al. (2016). In this previous study, we observed significant differences in the percentage of survivors and development time between both lines when reared on a standard medium. The heterogeneous population (experimental) was created by mixing the eight isofemale lines altogether as described in The *Drosophila* Synthetic Population Resource (DSPR, 2021). In brief, crossing between the newly emerged males of four isofemale lines and the females of the other four isofemale lines were performed. In parallel, another crossing was performed using the females of the first four lines and the males of the second four lines. The offspring of both crossings were thereafter mixed and made the experimental population (Exp). Exp was also reared in cages of 200 individuals of mixed sex under aseptic conditions. All homogenous and heterogeneous populations were at least reared in 3–5 replicates to prevent pseudo‐replication. For sample collection, one or two vials containing hard agar (double amount of agar) were used to collect eggs from each cage/population for the experimentation (Figure 2).

### 2.2 The bacterial pathogen

The bacterial pathogen *P*. *carotovorum* (CFBP 2141) was used as a pathogen of *D*. *melanogaster* larvae (Vodovar et al., 2004). To culture the bacterium, *P*. *carotovorum* stock was inoculated into LB broth and incubated in a rotatory incubator (Daihan Scientific, South Korea) at 30 ± 2°C and 200 rpm overnight. The resulting culture was harvested using a sterile vial containing Ringer's solution. The concentration of the bacterial suspension was determined by measuring the optical density (OD) with a spectrophotometer (Janeway, USA) at a wavelength of 600 nm (Elkayal et al., 2016).

### 2.3 Experimental design

To assess the effects of protein‐restricted diet and/or infection by *P*. *carotovorum* on the response of the populations A and B (genotypes) and the heterogeneous population (Exp), first instar *Drosophila* larvae were allowed to develop under four environmental conditions. These conditions were as follows: (i) standard medium in the absence of *P*. *carotovorum* (uninfected standard), (ii) restricted dietary medium in the absence of *P*. *carotovorum* (uninfected restricted), (iii) *P*. *carotovorum* + standard medium (infected standard), and (iv) *P*. *carotovorum* + restricted dietary medium (infected restricted). The restricted diet was identical to the standard diet but with a reduced concentration of yeast (10 g/L) (Elkayal et al., 2016). An infection condition was created by inoculating the media vials (10 ml size contains 3 ml medium) with 100 µl of *P*. *carotovorum* suspension, at an OD_600_ of 100. Control media were inoculated with the same volume of Ringer's solution. The media vials were incubated for 24 h at 30 ± 2°C to confirm the growth of the bacterium before the start of the experiments. The experimental design is summarized in Figure 2.

### 2.4 Adult emergence assay

Adult emergence is a major life‐history trait in insects and is closely related to fitness (Kristensen et al., 2016). To assess the effects of diet restriction and infection with *P*. *carotovorum* on adult emergence, the A and B populations (homogeneous) and the heterogeneous population were allowed to develop from the 1st instar until adult emergence in the four environmental conditions previously mentioned (10 larvae from each vial per population/condition). The emergence percentage was calculated as the number of adults that emerged out of 10 tested larvae in each replicate (a total of 70 larvae). The emergence was observed daily for 15 successive days after treatment. Five cages for every population were sampled twice. If an infestation or infection appeared, the cages/vials were discarded. This procedure was repeated seven times per population/condition.

### 2.5 Preparation of larval *D. melanogaster* whole‐body homogenate

To detect the effect of dietary restriction and bacterial infection on the fat and protein content as well as on the pathogen resistance of *Drosophila* larvae, 20–30 late 3rd‐instar larvae (5 days old) weighed (~50 mg) from each population and each treatment was homogenized in 500 µl of PBS (0.01 M, pH 7.4) on ice and centrifuged (Centurion Scientific, UK) for 10 min at 2000 × *g*. The supernatant was transferred into a new Eppendorf and stored at −20°C for later use. All the subsequent tests were repeated five times per population/condition.

### 2.6 Estimation of metabolic reserves in *D. melanogaster* populations in response to dietary restriction and bacterial infection

Whole‐body protein and lipid concentrations from homogeneous populations A and B as well as the heterogeneous population exposed to the four environmental conditions (uninfected standard, uninfected restricted, infected standard, and infected restricted) were determined spectrophotometrically. The protein content of the supernatants was determined using the Biuret method of Koller and Kaplan (1984) against standard protein albumin at 540 nm according to the instructions of the kit manufacturer (Bio diagnostics^®^, Giza, Egypt). The lipid content was also determined using the method of Zöllner and Kirsch (1962) at 520 nm against olive oil as a standard according to the instructions of the kit manufacturer (Bio diagnostics^®^, Giza, Egypt).

### 2.7 Measurement of immunity in *D. melanogaster* populations in response to dietary restriction and bacterial infection

To evaluate the level of immunity of *D*. *melanogaster* larvae to dietary restriction and bacterial infection, phenol‐oxidase (PO) and antibacterial activities were measured in the whole‐body homogenate.

PO activity was determined spectrophotometrically by measuring the formation of dopachrome according to the method of Ashida and Soederhaell (1984) with a slight modification. Aliquots (50 μl) of whole‐body homogenate (10 late 3rd instar larvae) were added to 350 µl of ice‐cold PBS and 400 µl of 20 mM l‐DOPA (Sigma, Germany), subsequently incubated for 20 min at 25 ± 2°C and measured at 490 nm against a blank (buffer + l‐DOPA). PO activity is expressed as units of PO/ml larval homogenate, where one unit is the amount of enzyme required to increase the absorbance at 490 nm by 0.001 min^−1^. Specific activity was calculated by dividing the enzyme activity of a specific volume by the protein content (mg) determined.

The antibacterial rate was measured using the whole‐body homogenate of *D*. *melanogaster* larvae according to the method of Haine et al. (2008) with slight modifications. Briefly, *P*. *carotovorum* suspension was prepared, adjusted to a concentration of 200 colony forming unit (CFU/ml), 50 µl of the whole‐body homogenate was added to 950 µl of the bacterial suspension, and the resulting solution was incubated in a rotary incubator at 30 ± 2°C and 200 rpm for 2 h. The final solution was diluted with 9 ml of Ringer's solution, and the OD was measured spectrophotometrically at 600 nm. The control contained 50 µl of Ringer's solution instead of whole‐body homogenate. The antibacterial rate was expressed as a rate, the number of CFU after 2 h of exposure to the whole‐body homogenate of *Drosophila* larvae in relation to the control measurement.

### 2.8 Statistical analyses

Data were expressed as mean ± standard deviation and checked for normality using the Shapiro–Wilk test. Adult emergence and antibacterial rate data were arcsine square‐root transformed. The effect of population, environmental changes (diet and infection) on adult emergence, metabolism (protein and lipid contents), and resistance (PO and antibacterial activities) of *D*. *melanogaster* were tested using a mixed‐effects model, where replicate (subject) was considered a random effect. Post hoc analyses were performed using the Bonferroni test for multiple comparisons to elucidate the effect of genotype and environmental change in GraphPad Prism version 8.0.2 for Windows, GraphPad Software, San Diego, California USA, http://www.graphpad.com. To quantify the extent of plasticity among isofemale lines (genotypes) and the heterogeneous population, relative distance plasticity index (RDPI), the difference between the trait values of the same genotype in different environments divided by their sum was calculated (Valladares et al., 2006). To compare the plasticity indices among the genotypes and the heterogeneous population, the GENMOD procedure was adopted. Normal distribution and identity link‐function were used in SAS v. 9.1 (SAS Institute, Inc., Cary, NC, USA).

## 3 RESULTS

### 3.1 Drosophila adult emergence in response to environmental changes

**FIGURE 3:**
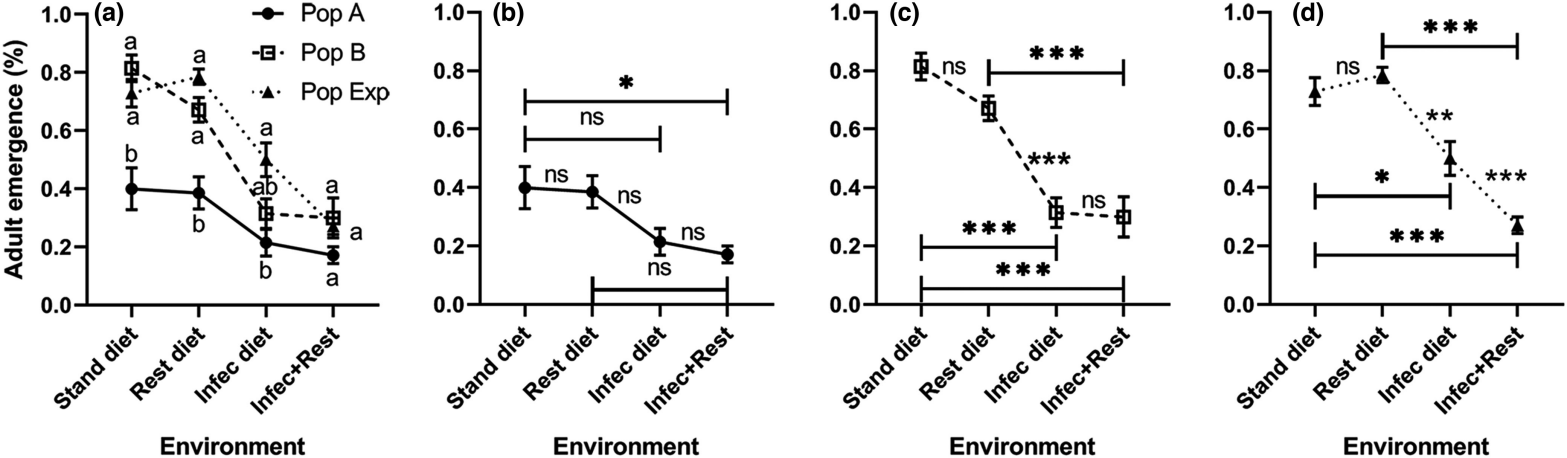
Adult emergence (mean ± SEM) of homogeneous (A and B) and heterogeneous (Exp) populations of *Drosophila melanogaster* raised under four different conditions: standard (stand) and restricted (rest) diets, bacteria‐infected (infec) and restricted + infected conditions. Data were analyzed using mixed‐effects model. There are significant differences among populations, environments and their interactions when *p* < .05. The population means with the same small letter are not significantly different in the same environment when *p* ≥ .025 (a). Charts (b), (c), and (d) show the response of populations A, B, and experimental alone among all environments. ns, *, ** and *** refer to non‐significant, and significant differences among environments when *p* ≥ .017, *p* < .017, .01, and .001 (multiple comparisons). *n* = seven replicates

To investigate the effect of protein‐restricted diet and/or exposure to the bacterium *P*. *carotovorum* on the survivorship of *D*. *melanogaster*, 1st instar larvae were allowed to develop until adult emergence in four environmental conditions: (uninfected standard), (uninfected restricted), (infected standard), and (infected restricted). Statistical analysis showed that the adult emergence (%) of *D*. *melanogaster* shows significant differences among populations (*F* (2, 72) = 32.23, *p* < .0001), environments (*F* (3, 72) =41.02, *p* < .0001), and their interaction (*F* (6, 72) = 3.26, *p* = .007) (Table S1). On either standard or restricted diet, the percentage emergence of the isofemale B and the experimental population (Exp) was significantly (*p* < .025) higher than that in population A. On the infected diet, only the Exp population displayed a higher (*p* < .025) emergence than that of the population A. However, all populations exhibited similar occlusion rates on the combined effect of diet restriction and bacterial infection (Figure 3a). Otherwise, population A of *D*. *melanogaster* did not show significant changes in the survivorship on infection or diet restriction. It showed a significant (*p* < .017) decrease in adult emergence only when raised under a combined effect of diet restriction and bacterial infection compared with that in the standard diet (Figure 3b). However, population B and Exp population showed significantly (*p* < .017) lower adult emergence on the infection and combined effect (Figure 3c, d). Exp population demonstrated a wider variability in adult emergence as it showed significant differences when those exposed to the single effect with that facing the combination of infection and diet restriction (Figures 3c and 8). Overall, these results indicated that the survivorship of different populations may evoke different effects based on genotype. However, most of the populations may be affected by extreme changes in the environment such as infection or more complex situations where multi‐change is involved.

### 3.2 *Drosophila* protein and lipid contents exhibit variation toward most environmental changes tested

To understand the effect of protein‐restricted diet and/or infection with *P*. *carotovorum* on the energy reserves of *D*. *melanogaster*, larvae of A, B, and Exp populations were raised under 4 different environmental conditions. The total body protein and lipid contents were determined in the late 3rd instar larvae.

#### 3.2.1 Protein content

**FIGURE 4:**
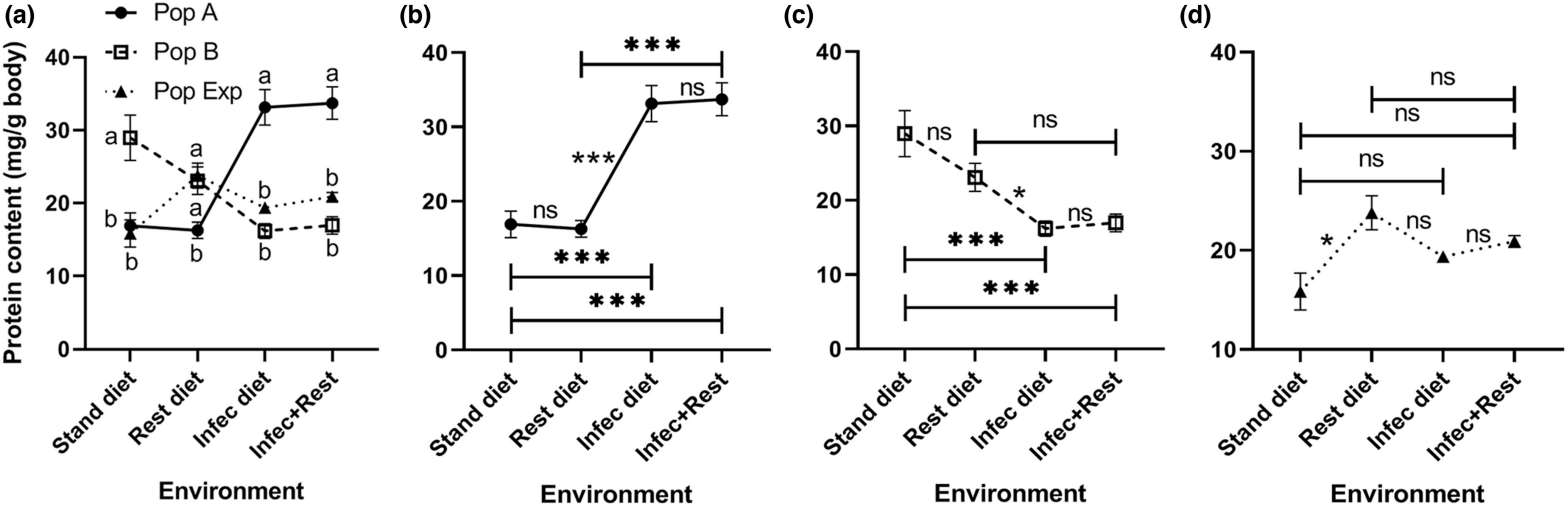
Whole‐body protein content (mean ± SEM) of homogeneous (A and B) and heterogeneous (Exp) populations of *Drosophila melanogaster* larvae raised under four different conditions: standard (stand) and restricted (Rest) diets, bacteria‐infected (Infec) and restricted + infected conditions. Data were analyzed using the mixed effects model. There are significant differences among populations and the interaction between population and the environment when *p* < .05. The population means with the same small letter are not significantly different in the same environment when *p* ≥ .025 (a). Charts (b), (c), and (d) show the response of populations A, B, and experimental alone among all environments. ns, * and *** refer to non‐significant, and significant differences among environments when *p* ≥ .017, *p* < .017 and .001 (multiple comparisons). *n* = five replicates

Our results showed that the total body protein content of *D*. *melanogaster* larvae depends on the genotype (*F* (2, 12) = 7.395, *p* = .0081) and the interaction with the environment (*F* (6, 36) = 22.67, *p* < .0001), as it differs significantly due to the effect of the population, whereas some populations display environmental changes in the protein content (Table S1). Multiple comparisons test in Figure 4a indicated that the protein content of the population B was significantly (*p* < .025) higher than those in A or Exp on the standard diet. On the restricted diet, B and Exp populations exhibited a significantly (*p* < .025) higher protein content than that in population A. Otherwise, B and Exp populations demonstrated significantly (*p* < .025) lower protein contents than that in population A when raised on an infected diet or combined stress of diet restriction and infection (Figure 4a). Population A exhibited significantly (*p* < .017) higher protein content upon exposure to infection with *P*. *carotovorum* or a combined effect of infection and diet restriction (Figure 4b). On the contrary, population B showed significantly (*p* < .017) lower protein content in those larvae raised in the infection or infection plus restriction diet compared to that raised on the standard diet (Figure 4c). In the heterogeneous population, only the larvae raised on diet restriction showed significantly (*p* < .017) higher protein content compared with those raised on a standard diet (Figure 4d). The heat map matrix for protein content of *D*. *melanogaster* larvae indicates phenotypic variation (plasticity) between A and B genotypes in the environments tested (Figure 8). The Exp population exhibited variability in protein content upon diet restriction (Figure 8). Based on the observed significant differences in the interaction between populations and environments for the *Drosophila* trait and the crossing reaction norms for the genotypes A and B (Figures 4b, c and 8), the protein contents of *D*. *melanogaster* reared in a standard diet exhibited a genotype‐by‐environment interaction upon exposure to the bacterium, *P*. *carotovorum*.

#### 3.2.2 Lipid content

**FIGURE 5:**
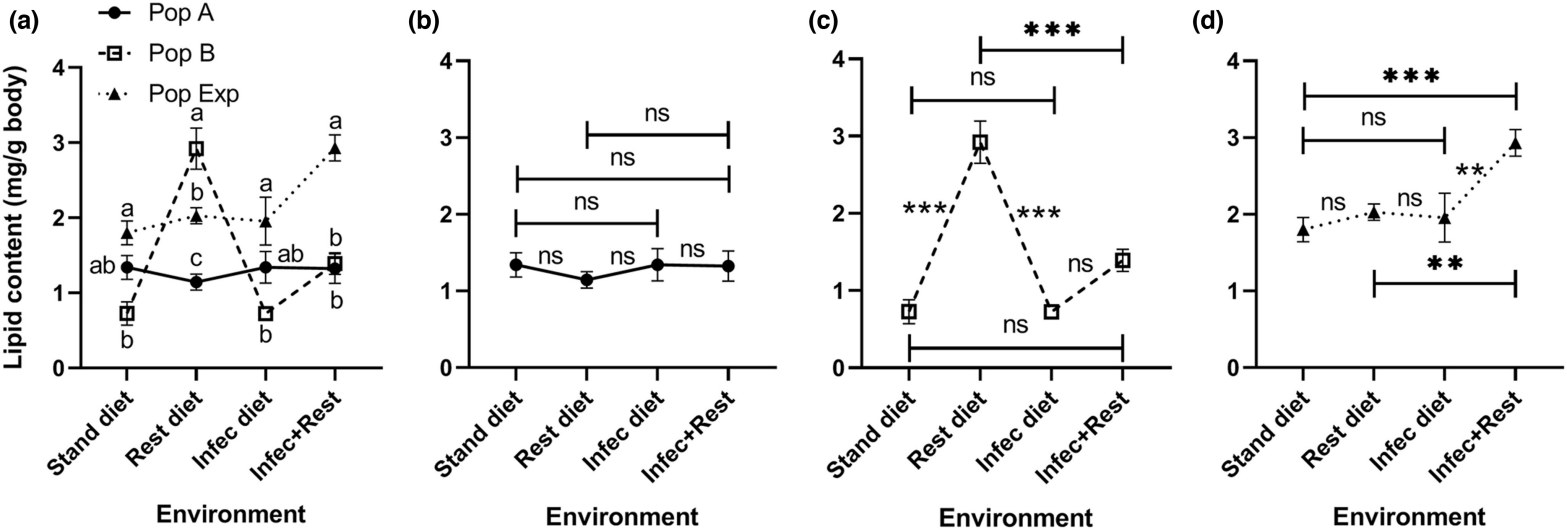
Whole‐body lipid content (mean ± SEM) of homogeneous (A and B) and heterogeneous (Exp) populations of *Drosophila melanogaster* larvae raised under four different conditions: standard (stand) and restricted (Rest) diets, bacteria‐infected (Infec) and restricted + infected conditions. Data were analyzed using the mixed‐effects model. There are significant differences among populations, environments, and their interactions when *p* < .05. The population means with the same small letter are not significantly different in the same environment when *p* ≥ .025 (a). Charts (b), (c), and (d) show the response of populations A, B, and experimental alone among all environments. ns, ** and *** refer to non‐significant, and significant differences among environments when *p* ≥ .017, .01, and .001 (multiple comparisons). *n* = five replicates

The lipid content of *D*. *melanogaster* larvae was affected by the population (*F* (2, 12) = 24.74, *p* < .0001), environment (*F* (3, 36) = 12.75, *p* < .0001), and their interaction (*F* (6, 36) = 13.77, *p* < .0001) (Figure 5). Notably, the responses of populations tested were diverse based on the type of environment (Table S1). On either standard or infected diet, population B showed significantly (*p* < .025) lower lipid content than that in the Exp population. On the restricted diet, the lipid contents of populations B and Exp were significantly (*p* < .025) higher than those of population A (multiple comparisons in Figure 5a). On double challenges with diet restriction and infection, the Exp population showed significantly (*p* < .025) higher lipids than those in populations A and B (Figure 5a). Comparison among environments demonstrated that changes could not induce a marked change in the lipid content in population A (Figure 5b). Population B showed significantly higher (*p* < .017) lipid content when raised on a restricted diet compared with all environments tested (Figure 5c). Similarly, the Exp population responded to the combined effect of diet restriction and infection by a marked (*p* < .017) increase in the lipid contents of larvae (Figure 5d). The heat map matrix of lipid contents shows that *D*. *melanogaster* genotypes A and B reared in different environments exhibited a restricted variation (plasticity) mainly owing to the observed changes in the B isofemale lines in response to diet restriction (Figure 8).

### 3.3 Drosophila immunity exhibits variation in all environmental changes tested

To determine the effects of diet restriction and/or infection with *P*. *carotovorum* on the host immunity, PO and antibacterial activities were measured in the whole‐body homogenate of *D*. *melanogaster* larvae.

#### 3.3.1 PO activity

**FIGURE 6:**
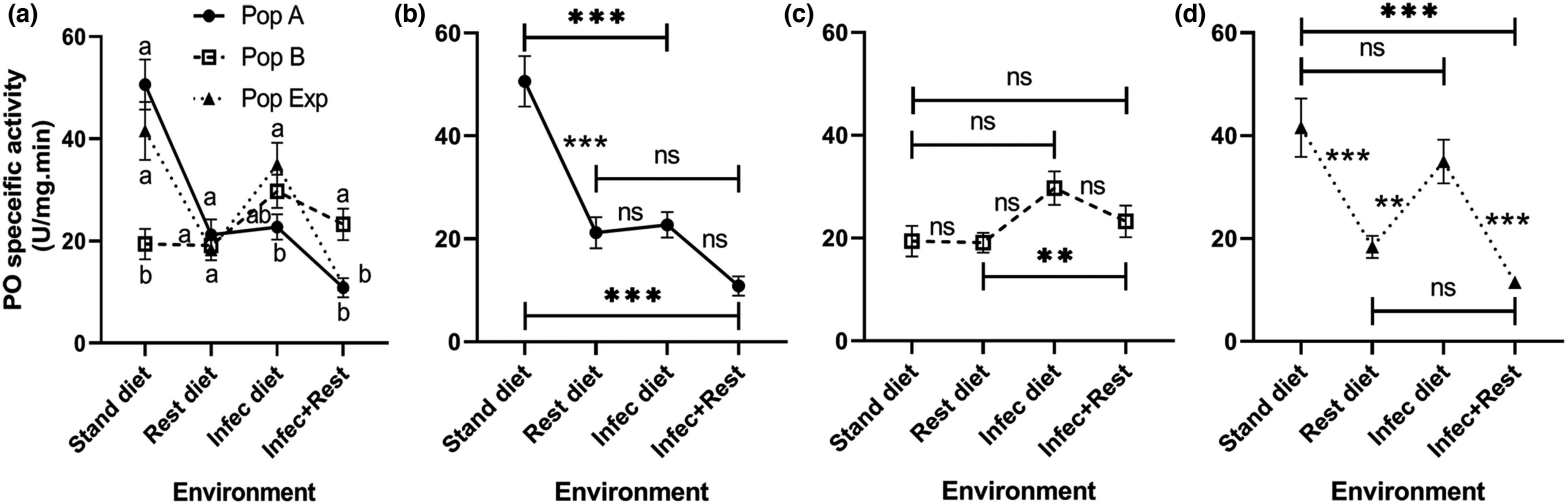
Phenol‐oxidase specific activity (mean ± SEM) of homogeneous (A and B) and heterogeneous (Exp) populations of *Drosophila melanogaster* larvae raised under four different conditions: standard (stand) and restricted (Rest) diets, bacteria‐infected (Infec) and restricted + infected conditions. Data were analyzed using the mixed effects model. There are significant differences among environments and the interaction between the environment and population when *p* < .05. The population means with the same small letter are not significantly different in the same environment when *p* ≥ .025 (a). Charts (b), (c), and (d) show the response of populations A, B, and experimental alone among all environments. ns, ** and *** refer to non‐significant, and significant differences among environments when *p* ≥ .017, .01, and .001 (multiple comparisons). *n* = five replicates

Figure 6 shows PO activities of all *D*. *melanogaster* populations tested under environmental changes in diet and treatment. Statistical analysis indicated that PO of *Drosophila* larvae is influenced by the environment (*F* (3, 36) = 29.58, *p* < .0001) and the interaction between environment and population (*F* (6, 36) = 11.14, *p* < .0001). On a standard diet, population B had the lowest (*p* < .025) PO activity among the populations tested. However, on the combined effect of diet restriction and bacterial infection, it had the highest (*p* < .025) PO activity among populations (Figure 6a). Otherwise, all populations exhibited similar PO activities on diet restriction. However, the population Exp exhibited a significantly (*p* < .025) higher PO response on the infection, while it had a lower (*p* < .025) response on the combined challenge of diet restriction and infection (Figure 6a). Populations A and Exp showed significant (*p* < .017) differences among the environments tested (Figure 6b, d). However, population B did not show any significant changes in PO on environmental changes (Figure 6c). In general, the heat map matrix indicates that PO activity in *Drosophila* larvae exhibited variation in most environments tested (Figure 8).

#### 3.3.2 Antibacterial rate

**FIGURE 7:**
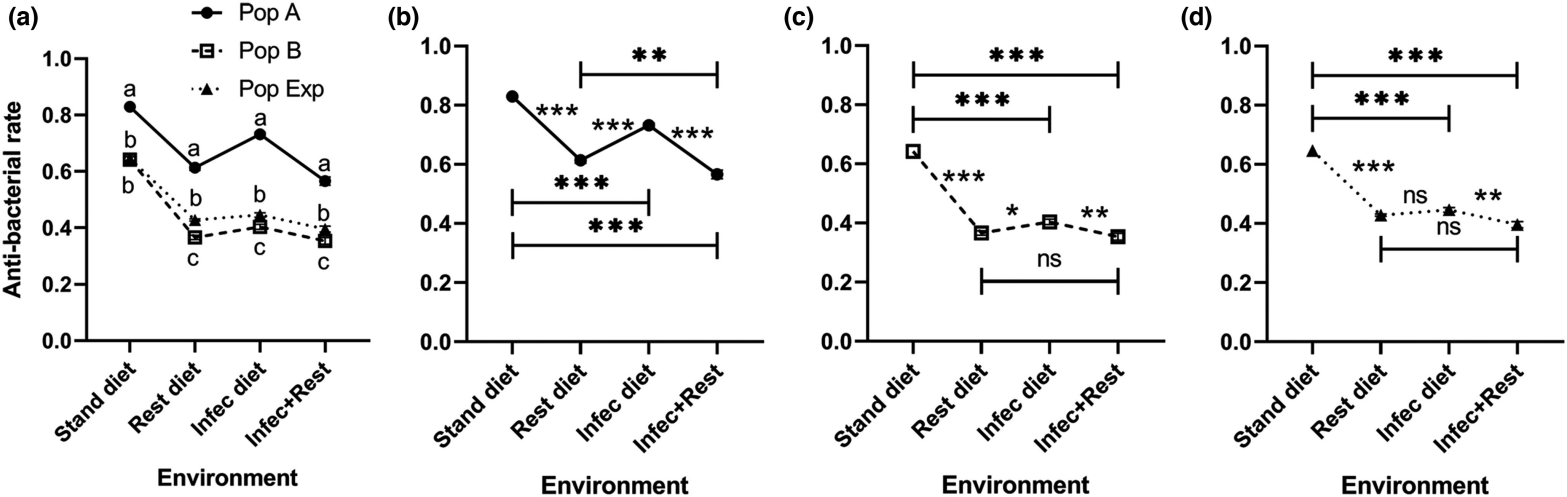
Antibacterial rate (mean ± SEM) of homogeneous (A and B) and heterogeneous (Exp) populations of *Drosophila melanogaster* larvae raised under four different conditions: standard (stand) and restricted (Rest) diets, bacteria‐infected (Infec), and restricted + infected conditions. Data were analyzed using the mixed effects model. There are significant differences among the populations, environments, and their interaction when *p* < .05. The population means with the same small letter are not significantly different in the same environment when *p* ≥ .025 (a). Charts (b), (c), and (d) show the response of populations A, B, and experimental alone among all environments. ns, *, ** and *** refer to non‐significant, and significant differences among environments when *p* ≥ .017, *p* < .017, .01, and .001 (multiple comparisons). *n* = five replicates

**FIGURE 8:**
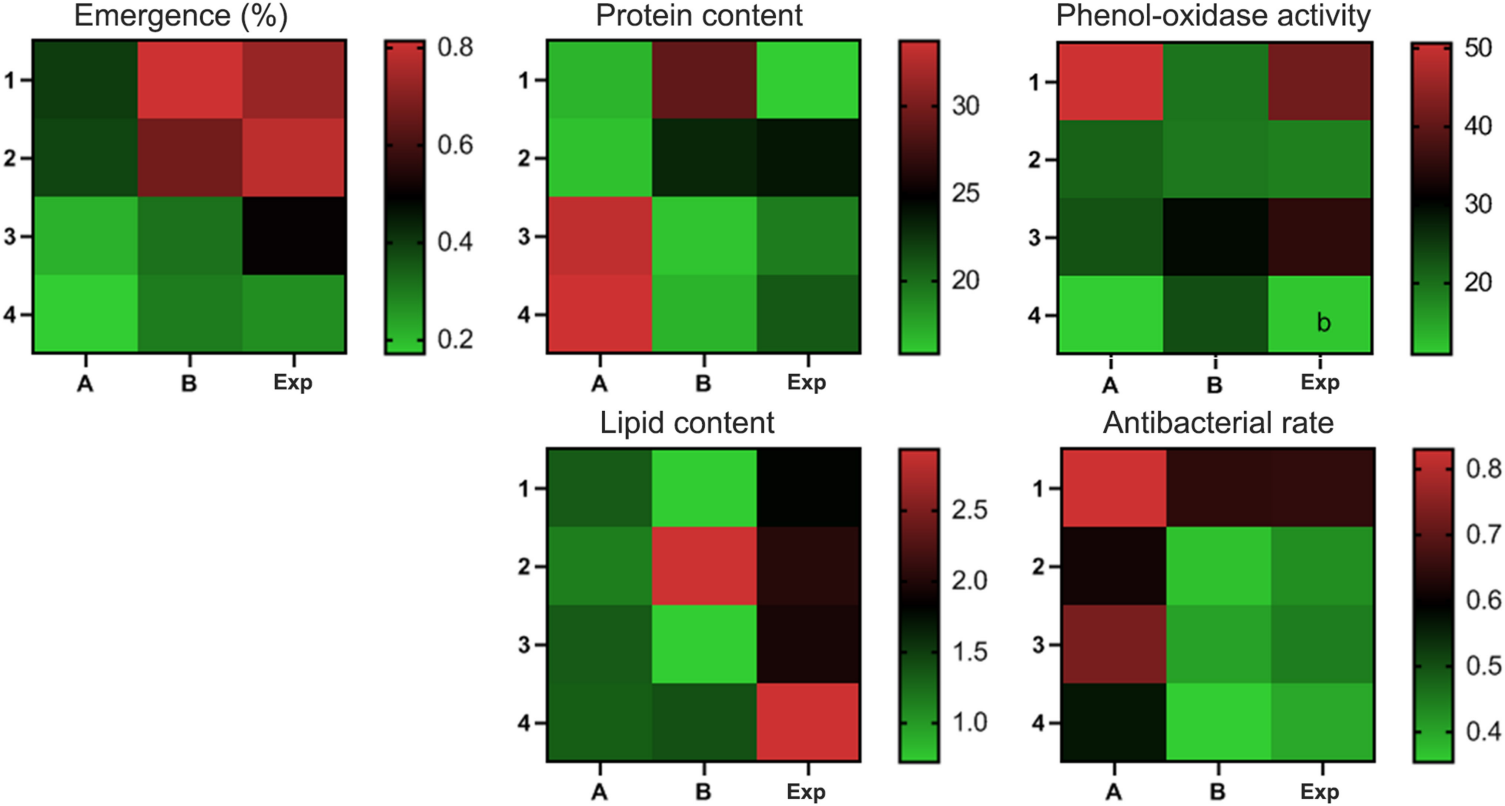
Heat map matrix for the response of the homogenous populations (A and B) compared with the heterogeneous population (Exp) across all the environments tested. The *X*‐axis displays the populations, while the environments: (1) standard diet at aseptic condition, (2) restricted diet with aseptic condition, (3) standard diet with infection, and (4) restricted diet with infection. The legend shows the low response in green, high in red and intermediate in the mixture

Larvae of *D*. *melanogaster* exhibited significantly different levels of antibacterial activities depending on population (*F* (2, 12) = 672.7, *p* < .0001) and environment (*F* (3, 36) = 551.4, *p* < .0001) and their interaction (*F* (6, 36) = 13.67, *p* < .0001) (Table S1). On the standard diet, population A showed a significantly (*p* < .025) higher antibacterial rate compared to population B or Exp. On the restricted or infected diets as well as the combined challenge, all populations showed significant (*p* < .025) differences in the antibacterial rate to each other in the following order (A > Exp > B) (Figure 7a). All populations tested exhibited different (*p* < .017) antibacterial rates with the environmental changes (Figure 7b–d). The highest antibacterial rate in all populations tested was observed on the standard diet. The populations showed antibacterial rates that change between diets as follows (standard > infected > restricted > infected plus restricted) (Figures 7 and 8). The heat map matrix for the antibacterial rate of *Drosophila* genotypes appeared to be variable in response to changes in diet and treatment conditions (Figure 8).

### 3.4 Quantification of the plasticity level among *Drosophila* homogenous and heterogeneous populations tested

Table 1 shows the values of RDPI calculated in the homogenous populations (A and B) and heterogeneous population (Exp) as well as the results of GENMOD analysis among them. Surprisingly, there is no significant difference in the plasticity of the tested populations regarding survivorship or antimicrobial rate. However, the order of plasticity in survivorship (A ˃ B ˃ Exp) was inversed in the antimicrobial rate for the genotypes. For the protein and lipid contents as well as PO activity, it was clear that plasticity (*p* < .05) differs among the populations. Multiple comparisons demonstrated that a genotype A has significantly (*p* < .025) higher plasticity in the protein reserves than the heterogeneous population Exp. The genotype B had higher (*p* < .025) plasticity in lipid reserves than those in both genotype A and the heterogeneous population Exp. The PO activity of genotype A and population Exp had higher (*p* < .025) plasticities than that of genotype B.

**TABLE 1:** Plasticity indices of the traits investigated in the homogenous (A and B) and heterogeneous (Exp) populations due to environmental changes (diet restriction and bacterial infection)

Trait	Plasticity index (RDPI) (mean ± SD)			*p*‐value of GENMOD
	A	B	Exp	
Survivorship	0.344 ± 0.217^a^	0.315 ± 0.219^a^	0.281 ± 0.192^a^	.5298
Protein content	0.221 ± 0.147^a^	0.162 ± 0.115^ab^	0.118 ± 0.086^b^	.0298
Lipid content	0.159 ± 0.097^b^	0.374 ± 0.201^a^	0.178 ± 0.134^b^	<.0001
PO specific activity	0.374 ± 0.121^b^	0.166 ± 0.128^a^	0.326 ± 0.174^b^	<.0001
Antibacterial rate	0.096 ± 0.050^a^	0.147 ± 0.113^a^	0.121 ± 0.079^a^	.1733

Note

Significant difference among the means of populations when *p* < .05. The population means with the same small letter are not significantly different when *p* ≥ .025 (Bonferroni correction for multiple comparisons).

## 4 DISCUSSION

The present study aimed to explore the contribution of different genotypes present in a given population for the reaction to bacterial infection under two different dietary regimens, namely a control, fully nutritious diet, and dietary restriction characterized by reduced protein content. Protein restriction is common and considered a major risk factor for death and disease (Dalvi et al., 2018; Victora et al., 2008). Hence, another question is how the protein restriction may affect a population and subpopulation? To answer these questions, we used an artificial, heterogeneous population (Exp) created from eight different isofemale lines and thus mostly reflecting the original population derived from Zimbabwe. We compared this heterogeneous population with two of these eight founding isofemale lines, each of which showed an extreme response to an important life‐history trait, adult emergence under normal conditions.

Concerning emergence, we observed that the heterogeneous population reflects almost completely the situation that we also observed for the fit genotype (population B). It was interesting to observe that the different populations showed partly opposite responses to different environmental influences (infection and protein restriction), which again emphasizes the central importance of the genotype. For example, the unfit genotype (population A) showed a significant decline in adult emergence compared with both, the fit genotype (population B) and the heterogeneous population on standard and restricted diets. However, the specific genotypes (populations A and B) showed similar emergence in the stressed environments (infected or restricted + infected). The variability within the heterogeneous population was presumably able to rescue the survival in the infected diet. With changing environments, population A mostly showed almost similar emergence. However, the fit genotype population (B) showed a decline when exposed to an infection or the combination of both stressors. The difference in adult emergence between populations A and B could be attributed to the fact that genotypes respond differentially to nutrients. This fact received support from the nutrient‐gene interactions that determine the benefits and risks of diet, that is, the interaction between a particular allele and a specific dietary exposure may lead to benefits for certain conditions or diseases (Hesketh et al., 2006).

To dissect the factors that may contribute to survivorship (adult emergence), we evaluated the immune competence of the populations in terms of PO activity and the antibacterial rate in the same environments. PO has a role in non‐self recognition within the insect body and evokes both cellular and humoral immune reactions (Söderhäll & Aspán, 1993; Takehana et al., 2002). Antimicrobial peptides (AMPs) are expressed in the hemolymph by the fat bodies in systemic reaction or other tissues like gut‐lining cells and epidermis upon sensing bacteria in a local reaction. The transduction of the signal is mediated via the *Toll* or *Imd* pathways (Khush et al., 2001; Lemaitre & Hoffmann, 2007). This can be measured as an antibacterial activity or rate in *Drosophila* body homogenate.

In the current study, the antibacterial rate appeared to be influenced by population and environment. The unfit genotype (A) exhibited a higher rate against *P*. *carotovorum* than the fit genotype (B) and the heterogeneous population, possibly owing to epigenetic and transcriptional differences and the subsequent different expression levels of antimicrobial peptides or reactive oxygen species (ROS) production that drive phenotypic diversity (Ecker et al., 2018; Lemaitre & Hoffmann, 2007). Another explanation could be that population A has less amidase peptidoglycan recognition proteins (PGRPs) such as PGRP‐LB and PGRP‐SC that can convert Gram‐negative peptidoglycan (PGN) into non‐immuno‐stimulatory fragments. This amidase PGN helps to economize the host resources during infection or under stress (Bischoff et al., 2006; Zaidman‐Rémy et al., 2006). When the environment changes in terms of diet or treatment conditions, *Drosophila* larvae change appropriately. The antibacterial rates of the different genotypes showed a particularly interesting interdependence on infection or the protein content in the food. The response of the heterogeneous population almost completely followed the fit genotype. In the stressed environments tested, the unfit genotype exhibited higher antimicrobial activity than that in the fit genotype. So that in a few aspects, the two homogeneous populations (A and B) took extreme positions, for example in survivorship and antibacterial rate.

Surprisingly, the association between response types was different for another independent aspect of insect immunity, the PO activity. Here, the heterogeneous population showed changes in response to infection and protein restriction rather similarly to the unfit genotype than the fit one. In the present study, PO activity in *Drosophila* larvae exhibited a significant decrease in response to diet restriction and the presence of a bacterial pathogen.

The response of the heterogeneous population reflects the response of one homogenous subpopulation for one immune‐related trait, whereas it is close to the response type of the second homogenous subpopulation for the second immune‐related trait. This could be a reflection of the trade‐off between individual traits to economise the internal energy available (Rigby et al., 2002). The trade‐off is widely known between survival and fecundity in *Drosophila* (Chippindale et al., 2004). In bumblebees, Moret and Schmid‐Hempel (2000) detected a negative correlation between antibacterial and PO activities in response to lipopolysaccharide (LPS) treatment. We observed a reduced antibacterial rate and protein content in response to an infection in most populations tested. However, population A showed increased protein with infection. For protein‐restricted diet, or even malnutrition, this was expected (Joost et al., 2007). As in previous studies, the protein‐restricted diet was unable to fulfill the need of diseased insects to combat infections or build up the protein content (Lee et al., 2006; Thompson & Redak, 2000). It has been shown that nutritional deficiency affects the resistance of individuals to disease in general (Calder & Jackson, 2000). The differences observed in the responses of different subpopulations show that general fitness differences are present within a population (Elkayal et al., 2016; Meshrif & Elkholy, 2015) based on the nutrient–gene interactions in the subpopulations in health and disease (Hesketh et al., 2006). Host immunity is also a complex trait. Several intrinsic and extrinsic factors interact with each other to modulate the immune response (Ponton et al., 2011). In an intrinsic view, the metabolic reserves and host's microbiota had a major effect on mounting a suitable immune reaction to the pathogen (Wen et al., 2008). Therefore, this necessitates measuring the energy reserves like lipids as well as proteins. Both are the major substrate for producing immunological components such as PO and antimicrobial peptides and could affect the immune response and in turn survival (Djawdan et al., 1998; Lee et al., 2006; Mullen & Goldsworthy, 2003).

The differential reaction of the major energy stores, protein, and lipids, with respect to the genotype, is not as obvious as observed for the two immune‐related traits. For the protein and lipid contents, the heterogeneous population sometimes showed similar contents to one of the genotypes based on environmental exposure. In a few of these responses, the reaction of the heterogeneous population is strictly intermediate if compared with the two genotypes A and B (homogenous populations), a behavior that might be expected because the heterogeneous population usually consists of different genotypes with diverse performances. Principally, the quantification of metabolic reserves such as protein and lipid contents may provide information about the physiological status of animals under experimental conditions (restricted diet and/or bacterial infection) (Ellers et al., 2011; Wilder et al., 2016) and may also be an indicator of how they might respond to major environmental changes. For example, the whole‐body proteins of the unfit and fit genotypes showed an opposite response to infection or double effect of infection and diet restriction. So that it was easy to observe that a protein‐restricted diet can exacerbate the incidence of infection as previously reported among humans (Calder & Jackson, 2000). Protein content demonstrated lower levels in the fit genotype (B) in response to infection or diet restriction plus infection, whereas the unfit genotype (A) exhibited a higher level if confronted with the same challenges. Based on this result, we infer that bacterial infection and the combined effect with a protein‐restricted diet contribute to metabolic phenotypes in *D*. *melanogaster* and that this relationship may be genotype‐dependent (Hesketh et al., 2006; Reed et al., 2010). A recent study reported that mortality in *Drosophila* could be attributed to the effect of macronutrient balance on the immune response (Ponton et al., 2020). It is accepted that the plasticity of a given trait could also change in response to environmental fluctuations to increase the survival of the genotype. Canalization may explain the variances among genotypes on an evolutionary scale (Hallgrimsson et al., 2019). In the present study, *D*. *melanogaster* exhibited a genotype‐by‐environment interaction for protein content in response to infection when reared on a standard diet. This result may explain why distinct genotypes vary in their phenotypes when exposed to specific environmental changes (Lazzaro et al., 2008).

In the present study, the whole‐body lipid content varied in *D*. *melanogaster* as a function of genotype, indicating that lipid reserves are very sensitive to the genetic makeup of individuals, in agreement with Reed et al. (2010). The fit genotype exhibited a higher level of lipid content only when exposed to a restricted diet, while the heterogeneous population had a higher response on exposure to a combined effect of diet restriction and bacterial infection. This may imply that the heterogeneous population was more resistant to the environmental changes than the homogenous fit population (B). However, the increase in lipid content of the fit genotype of a population may be the result of an acceleration of lipogenesis on sensing of danger or stresses reported previously (Gholizadeh et al., 2019; Priyadarsini et al., 2020), as lipids represent a pool of energy reserves that can be used in such conditions after carbohydrates (Thompson, 2003).

RDPI quantified in this study indicated phenotypic variation (plasticity) in the homogenous populations (A and B) corresponds to the variability detected in the heterogeneous population due to already genetic diversity among individuals. The observed reduction in variance in the heterogeneous population could be explained by individual (genotype) variation, which may, in turn, buffer the response against environmental perturbations (Debat & David, 2001). This suggests that even the homogenous populations have a chance to resist environmental stress even if their traits are not optimal (Chambel et al., 2005; Whitman & Agrawal, 2009).

In conclusion, trait plasticity may help homogeneous populations (genotypes) with less than optimal phenotypes to tolerate harsh environmental conditions before exhibiting a genetic change upon adaptation. A kind of trade‐off was observed between PO and antibacterial rate. In the future study, we would like to assess the evolution by artificial selection of such populations and the major adaptation for survival.

## AUTHOR CONTRIBUTIONS

**Wesam S. Meshrif:** Conceptualization (lead); Data curation (equal); Formal analysis (lead); Investigation (equal); Methodology (lead); Resources (equal); Supervision (equal); Visualization (lead); Writing – original draft (lead). **Sandy H. Elkayal:** Investigation (equal); Methodology (supporting); Writing – original draft (supporting). **Mohamed A. Soliman:** Resources (equal); Supervision (equal); Writing – review & editing (supporting). **Amal I. Seif:** Conceptualization (supporting); Methodology (supporting); Resources (equal); Supervision (equal); Writing – review & editing (supporting). **Thomas Roeder:** Conceptualization (supporting); Data curation (equal); Writing – review & editing (lead).

## CONFLICT OF INTEREST

The authors declare that they have no conflict of interest.

## Supporting information

Table S1

## Data Availability

The data that support the findings of this study is openly available in [Dryad] at https://doi.org/10.5061/dryad.4mw6m90cs.

## References

[ece38960-bib-0001] Agrios, G. (1997). Plant pathology, 4th ed. (pp. 619). Academic Press.

[ece38960-bib-0002] Ashburner, M. (1989). Drosophila: A laboratory handbook. Cold Spring Harbor Laboratory Press.

[ece38960-bib-0003] Ashida, M. , & Soederhaell, K. (1984). The prophenoloxidase activating system in crayfish. Comparative Biochemistry & Physiology. B. Comparative Biochemistry, 74, 21–26.

[ece38960-bib-0004] Basset, A. , Khush, R. S. , Braun, A. , Gardan, L. , Boccard, F. , Hoffmann, J. A. , & Lemaitre, B. (2000). The phytopathogenic bacteria *Erwinia carotovora* infects *Drosophila* and activates an immune response. Proceedings of the National Academy of Sciences, 97, 3376–3381. 10.1073/pnas.070357597 PMC16247 10725405

[ece38960-bib-0005] Bischoff, V. , Vignal, C. , Duvic, B. , Boneca, I. G. , Hoffmann, J. A. , & Royet, J. (2006). Downregulation of the *Drosophila* immune response by peptidoglycan‐recognition proteins SC1 and SC2. PLoS Path, 2, e14. 10.1371/journal.ppat.0020014 PMC1383489 16518472

[ece38960-bib-0006] Boeing, H. (2013). Nutritional epidemiology: New perspectives for understanding the diet‐disease relationship? European Journal of Clinical Nutrition, 67, 424–429. 10.1038/ejcn.2013.47 23443832

[ece38960-bib-0007] Calder, P. C. , & Jackson, A. A. (2000). Undernutrition, infection and immune function. Nutrition Research Reviews, 13, 3–29. 10.1079/095442200108728981 19087431

[ece38960-bib-0008] Chambel, M. R. , Climent, J. , Alía, R. , & Valladares, F. (2005). Phenotypic plasticity: A useful framework for understanding adaptation in forest species. Forest Systems, 14, 334–344.

[ece38960-bib-0009] Chippindale, A. K. , Leroi, A. M. , Kim, S. B. , & Rose, M. R. (2004). Phenotypic plasticity and selection in *Drosophila* life‐history evolution. I. Nutrition and the cost of reproduction. In M. R. Rose, H. B. Passananti, & M. Matos (Eds.), Methuselah flies: A case study in the evolution of aging (pp. 122–144). World Scientific.

[ece38960-bib-0010] Cotter, S. C. , Reavey, C. E. , Tummala, Y. , Randall, J. L. , Holdbrook, R. , Ponton, F. , Simpson, S. J. , Smith, J. A. , & Wilson, K. (2019). Diet modulates the relationship between immune gene expression and functional immune responses. Insect Biochemistry and Molecular Biology, 109, 128–141. 10.1016/j.ibmb.2019.04.009 30954680 PMC6527921

[ece38960-bib-0011] Dalvi, P. S. , Yang, S. , Swain, N. , Kim, J. , Saha, S. , Bourdon, C. , Zhang, L. , Chami, R. , & Bandsma, R. H. J. (2018). Long‐term metabolic effects of malnutrition: Liver steatosis and insulin resistance following early‐life protein restriction. PLoS One, 13(7), e0199916. 10.1371/journal.pone.0199916 29965973 PMC6028108

[ece38960-bib-0012] Debat, V. , & David, P. (2001). Mapping phenotypes: Canalization, plasticity and developmental stability. Trends in Ecology & Evolution, 16, 555–561. 10.1016/S0169-5347(01)02266-2

[ece38960-bib-0013] Debat, V. , & Le Rouzic, A. (2019). Canalization, a central concept in biology. Seminars in Cell & Developmental Biology, 88, 1–3. 10.1016/j.semcdb.2018.05.012 29775661

[ece38960-bib-0014] Djawdan, M. , Chippindale, A. K. , Rose, M. R. , & Bradley, T. J. (1998). Metabolic reserves and evolved stress resistance in *Drosophila melanogaster* . Physiological Zoology, 71(5), 584–594. 9754535 10.1086/515963

[ece38960-bib-0015] DSPR: The Drosophila Synthetic Population Resource (2021) . http://wfitch.bio.uci.edu/~dspr/Tools/Designs/index.html

[ece38960-bib-0016] Ecker, S. , Pancaldi, V. , Valencia, A. , Beck, S. , & Paul, D. S. (2018). Epigenetic and transcriptional variability shape phenotypic plasticity. BioEssays, 40, 1700148. 10.1002/bies.201700148 29251357

[ece38960-bib-0017] Elkayal, S. H. , Meshrif, W. S. , Soliman, M. A. , & Seif, A. I. (2016). Fitness of *Drosophila melanogaster* (Diptera: Drosophilidae) following bacterial infection under influence of two different diet regimes and host heterogeneity. African Entomology, 24, 476–488.

[ece38960-bib-0018] Ellers, J. , Ruhe, B. , & Visser, B. (2011). Discriminating between energetic content and dietary composition as an explanation for dietary restriction effects. Journal of Insect Physiology, 57, 1670–1676. 10.1016/j.jinsphys.2011.08.020 21914451

[ece38960-bib-0019] Flatt, T. (2020). Life‐history evolution and the genetics of fitness components in *Drosophila melanogaster* . Genetics, 214, 3–48. 31907300 10.1534/genetics.119.300160 PMC6944413

[ece38960-bib-0020] Gholizadeh, P. , Mahallei, M. , Pormohammad, A. , Varshochi, M. , Ganbarov, K. , Zeinalzadeh, E. , Yousefi, B. , Bastami, M. , Tanomand, A. , Mahmood, S. S. , Yousefi, M. , Asgharzadeh, M. , & Kafil, H. S. (2019). Microbial balance in the intestinal microbiota and its association with diabetes, obesity and allergic disease. Microbial Pathogenesis, 127, 48–55. 10.1016/j.micpath.2018.11.031 30503960

[ece38960-bib-0021] Haine, E. R. , Moret, Y. , Siva‐Jothy, M. T. , & Rolff, J. (2008). Antimicrobial defense and persistent infection in insects. Science, 322, 1257–1259. 10.1126/science.1165265 19023083

[ece38960-bib-0022] Hallgrimsson, B. , Green, R. M. , Katz, D. C. , Fish, J. L. , Bernier, F. P. , Roseman, C. C. , Young, N. M. , Cheverud, J. M. , & Marcucio, R. S. (2019). The developmental‐genetics of canalization. Seminars in Cell & Developmental Biology, 88, 67–79. 10.1016/j.semcdb.2018.05.019 29782925 PMC6251770

[ece38960-bib-0023] Hawley, D. M. , & Altizer, S. M. (2011). Disease ecology meets ecological immunology: Understanding the links between organismal immunity and infection dynamics in natural populations. Functional Ecology, 25, 48–60. 10.1111/j.1365-2435.2010.01753.x

[ece38960-bib-0024] Hesketh, J. , Wybranska, I. , Dommels, Y. , King, M. , Elliott, R. , Pico, C. , & Keijer, J. (2006). Nutrient–gene interactions in benefit–risk analysis. British Journal of Nutrition, 95(6), 1232–1236. 10.1079/BJN20061749 16768850

[ece38960-bib-0025] Howick, V. M. , & Lazzaro, B. P. (2014). Genotype and diet shape resistance and tolerance across distinct phases of bacterial infection. BMC Evolutionary Biology, 14, 56. 10.1186/1471-2148-14-56 24655914 PMC3997931

[ece38960-bib-0026] Joost, H.‐G. , Gibney, M. J. , Cashman, K. D. , Görman, U. , Hesketh, J. E. , Mueller, M. , van Ommen, B. , Williams, C. M. , & Mathers, J. C. (2007). Personalised nutrition: Status and perspectives. British Journal of Nutrition, 98, 26–31. 10.1017/S0007114507685195 17381877

[ece38960-bib-0027] Khush, R. S. , Leulier, F. , & Lemaitre, B. (2001). Drosophila immunity: Two paths to NF‐κB. Trends in Immunology, 22, 260–264. 10.1016/S1471-4906(01)01887-7 11323284

[ece38960-bib-0028] Koller, A. , & Kaplan, L. (1984). Total serum protein. In A. Pesce , & L. Kaplans (Eds.), Clinical chemistry, theory, analysis, and correlation (pp. 1316–1319). Mosby Company.

[ece38960-bib-0029] Kristensen, T. N. , Henningsen, A. K. , Aastrup, C. , Bech‐Hansen, M. , Bjerre, L. B. H. , Carlsen, B. , Hagstrup, M. , Jensen, S. G. , Karlsen, P. , Kristensen, L. , Lundsgaard, C. , Møller, T. , Nielsen, L. D. , Starcke, C. , Sørensen, C. , & Schou, M. F. (2016). Fitness components of *Drosophila melanogaster* developed on a standard laboratory diet or a typical natural food source. Insect Science, 23, 771–779. 25989059 10.1111/1744-7917.12239

[ece38960-bib-0030] Lazzaro, B. P. , Flores, H. A. , Lorigan, J. G. , & Yourth, C. P. (2008). Genotype‐by‐environment interactions and adaptation to local temperature affect immunity and fecundity in *Drosophila melanogaster* . PLoS Path, 4, e1000025. 10.1371/journal.ppat.1000025 PMC2265416 18369474

[ece38960-bib-0031] Lee, K. , Cory, J. , Wilson, K. , Raubenheimer, D. , & Simpson, S. (2006). Flexible diet choice offsets protein costs of pathogen resistance in a caterpillar. Proceedings of the Royal Society B: Biological Sciences, 273(1588), 823–829. 10.1098/rspb.2005.3385 PMC1560230 16618675

[ece38960-bib-0032] Lemaitre, B. , & Hoffmann, J. (2007). The host defense of *Drosophila melanogaster* . Annual Review of Immunology, 25, 697–743. 10.1146/annurev.immunol.25.022106.141615 17201680

[ece38960-bib-0033] Meshrif, W. S. , & Elkholy, S. E. (2015). Genotype and environment shape the fitness of *Drosophila melanogaster* . The Journal of Basic & Applied Zoology, 68, 1–9. 10.1016/j.jobaz.2015.01.003

[ece38960-bib-0034] Min, K.‐J. , Flatt, T. , Kulaots, I. , & Tatar, M. (2007). Counting calories in *Drosophila* diet restriction. Experimental Gerontology, 42, 247–251. 10.1016/j.exger.2006.10.009 17125951 PMC2606145

[ece38960-bib-0035] Moret, Y. , & Schmid‐Hempel, P. (2000). Survival for immunity: The price of immune system activation for bumblebee workers. Science, 290(5494), 1166–1168. 11073456 10.1126/science.290.5494.1166

[ece38960-bib-0036] Mullen, L. , & Goldsworthy, G. (2003). Changes in lipophorins are related to the activation of phenoloxidase in the haemolymph of *Locusta migratoria* in response to injection of immunogens. Insect Biochemistry and Molecular Biology, 33(7), 661–670. 10.1016/S0965-1748(03)00045-6 12826093

[ece38960-bib-0037] Oms, C. S. , Cerdá, X. , & Boulay, R. (2017). Is phenotypic plasticity a key mechanism for responding to thermal stress in ants? The Science of Nature, 104, 42. 10.1007/s00114-017-1464-6 28470449

[ece38960-bib-0038] Ponton, F. , Morimoto, J. , Robinson, K. , Kumar, S. , Cotter, S. , Wilson, K. , Simpson, S. J. (2020). Macronutrients modulate resistance to infection and immunity in *Drosophila* . Journal of Animal Ecology, 89(2), 460–470. 31658371 10.1111/1365-2656.13126 PMC7027473

[ece38960-bib-0039] Ponton, F. , Wilson, K. , Cotter, S. C. , Raubenheimer, D. , & Simpson, S. J. (2011). Nutritional immunology: A multi‐dimensional approach. PLoS Pathogenes, 7(12), e1002223. 10.1371/journal.ppat.1002223 PMC3228798 22144886

[ece38960-bib-0040] Priyadarsini, S. , Mukherjee, S. , Samikshya, S. N. , Bhanja, A. , Paikra, S. K. , Nayak, N. , & Mishra, M. (2020). Dietary infection of Enterobacter ludwigii causes fat accumulation and resulted in the diabetes‐like condition in Drosophila melanogaster. Microbial Pathogenesis, 149, 104276. 10.1016/j.micpath.2020.104276 32590093

[ece38960-bib-0041] Reed, L. K. , Williams, S. , Springston, M. , Brown, J. , Freeman, K. , Desroches, C. E. , Sokolowski, M. B. , & Gibson, G. (2010). Genetype‐by‐diet interactions drive metabolic phenotype variation in *Drosophila melanogaster* . Genetics, 185, 1009–1019. 20385784 10.1534/genetics.109.113571 PMC2907188

[ece38960-bib-0042] Rigby, M. C. , Hechinger, R. F. , & Stevens, L. (2002). Why should parasite resistance be costly? Trends in Parasitology, 18, 116–120. 10.1016/S1471-4922(01)02203-6 11854088

[ece38960-bib-0043] Rion, S. , & Kawecki, T. J. (2007). Evolutionary biology of starvation resistance: What we have learned from Drosophila. Journal of Evolutionary Biology, 20, 1655–1664. 17714282 10.1111/j.1420-9101.2007.01405.x

[ece38960-bib-0044] Schlichting, C. D. , & Pigliucci, M. (1998). Phenotypic evolution: A reaction norm perspective. Sinauer Associates Incorporated, Sunderland.

[ece38960-bib-0045] Schneider, D. (2000). Using *Drosophila* as a model insect. Nature Reviews Genetics, 1, 218–226. 10.1038/35042080 11252751

[ece38960-bib-0046] Söderhäll, K. , & Aspán, A. (1993). Prophenoloxidase activating system and its role in cellular communication. In J. P. N. Pathak (Ed.), Insect Immunity (pp. 113–129). Springer‐Science+Business Media. B.V.

[ece38960-bib-0047] Takehana, A. , Katsuyama, T. , Yano, T. , Oshima, Y. , Takada, H. , Aigaki, T. , & Kurata, S. (2002). Overexpression of a pattern‐recognition receptor, peptidoglycan‐recognition protein‐LE, activates imd/relish‐mediated antibacterial defense and the prophenoloxidase cascade in *Drosophila* larvae. Proceedings of the National Academy of Sciences, 99(21), 13705–13710. 10.1073/pnas.212301199 PMC129750 12359879

[ece38960-bib-0048] Thompson, S. N. (2003). Trehalose – The insect ‘Blood’ sugar. Advance in Insect Physiology, 31, 205–285.

[ece38960-bib-0049] Thompson, S. N. , & Redak, R. A. (2000). Interactions of dietary rotein and carbohydrate determine blood sugar level and regulate nutrient selection in the insect Manduca sexta L. Biochimica Et Biophysica Acta, 1523, 91–102. 11099862 10.1016/s0304-4165(00)00102-1

[ece38960-bib-0050] Valladares, F. , Sanchez‐Gomez, D. , & Zavala, M. A. (2006). Quantitative estimation of phenotypic plasticity: Bridging the gap between the evolutionary concept and its ecological applications. Journal of Ecology, 94, 1103–1116. 10.1111/j.1365-2745.2006.01176.x

[ece38960-bib-0051] Victora, C. G. , Adair, L. , Fall, C. , Hallal, P. C. , Martorell, R. , Richter, L. , Sachdev, H. S. (2008). Maternal and child undernutrition: Consequences for adult health and human capital. The Lancet, 371(9609), 340–357. 10.1016/S0140-6736(07)61692-4 PMC2258311 18206223

[ece38960-bib-0052] Vieira, F. J. D. (2014). The impact of the phytopathogen *Pectobacterium carotovorum* in *Drosophila melanogaster development* . M Sc. Biology. Universidade de Lisboa.

[ece38960-bib-0053] Vodovar, N. , Acosta, C. , Lemaitre, B. , & Boccard, F. (2004). *Drosophila*: A polyvalent model to decipher host‐pathogen interactions. Trends in Microbiology, 12, 235–242. 10.1016/j.tim.2004.03.007 15120143

[ece38960-bib-0054] Vrijenhoek, R. C. (1986). Host‐parasite coevolution: Ecology and genetics of host‐parasite interactions. Science, 232, 112. 10.1126/science.232.4746.112 17774013

[ece38960-bib-0055] Wen, L. , Ley, R. E. , Volchkov, P. Y. , Stranges, P. B. , Avanesyan, L. , Stonebraker, A. C. , Changyun, H. , Susan Wong, F. , Szot, G. L. , Bluestone, J. A. , Gordon, J. I. , & Chervonsky, A. V. (2008). Innate immunity and intestinal microbiota in the development of Type 1 diabetes. Nature, 455(7216), 1109–1113. 18806780 10.1038/nature07336 PMC2574766

[ece38960-bib-0056] Whitman, D. W. , & Agrawal, A. A. (2009). What is phenotypic plasticity and why is it important? In D. W. Whitman , & T. N. Ananthakrishnans (Eds.), Phenotypic plasticity of insects: Mechanisms and consequences (pp. 1–63). Science Publishers Enfield.

[ece38960-bib-0057] Wilder, S. M. , Raubenheimer, D. , & Simpson, S. J. (2016). Moving beyond body condition indices as an estimate of fitness in ecological and evolutionary studies. Functional Ecology, 30, 108–115. 10.1111/1365-2435.12460

[ece38960-bib-0058] Zaidman‐Rémy, A. , Hervé, M. , Poidevin, M. , Pili‐Floury, S. , Kim, M.‐S. , Blanot, D. , Oh, B.‐H. , Ueda, R. , Mengin‐Lecreulx, D. , & Lemaitre, B. (2006). The Drosophila amidase PGRP‐LB modulates the immune response to bacterial infection. Immunity, 24, 463–473. 10.1016/j.immuni.2006.02.012 16618604

[ece38960-bib-0059] Zargar, U. R. , Chishti, M. Z. , Ahmed, F. , & Rather, M. I. (2015). Does alternation in biodiversity really affect disease outcomes? ‐ A debate is brewing. Saudi Journal of Biological Sciences, 22, 14–18. 25561877 10.1016/j.sjbs.2014.05.004 PMC4281613

[ece38960-bib-0060] Zöllner, N. , & Kirsch, K. (1962). Colorimetric method for determination of total lipids. Journal of Experimental Medicine, 135, 545–550.

