# Supplementary material for: Metabolic and immunological responses of Drosophila melanogaster to dietary restriction and bacterial infection differ substantially between genotypes in a population
Source: Ecol Evol. 2022 May 24;12(5):e8960. doi: 10.1002/ece3.8960 (PMC9130643; doi:10.1002/ece3.8960)
Supplement: Supplementary file 1 — Table S1 [file ECE3-12-e8960-s001.docx]

Statistics of % emergence

| Table Analyzed | Transform of Transform of Data 1 survival |  |  |  |  |
| --- | --- | --- | --- | --- | --- |
|  |  |  |  |  |  |
| Mixed-effects model (REML) | Matching: Stacked |  |  |  |  |
| Assume sphericity? | Yes |  |  |  |  |
| Alpha | 0.05 |  |  |  |  |
|  |  |  |  |  |  |
| Fixed effects (type III) | P value | P value summary | Statistically significant (P < 0.05)? | F (DFn, DFd) |  |
| Environment | <0.0001 | **** | Yes | F (3, 72) = 41.02 |  |
| Population | <0.0001 | **** | Yes | F (2, 72) = 32.23 |  |
| Environment x Population | 0.0069 | ** | Yes | F (6, 72) = 3.258 |  |
|  |  |  |  |  |  |
| Random effects | SD | Variance |  |  |  |
| Subject | 0 | 0 |  |  |  |
| Residual | 0.1564 | 0.02446 |  |  |  |
|  |  |  |  |  |  |
| Was the matching effective? |  |  |  |  |  |
| Chi-square, df |  |  |  |  |  |
| P value |  |  |  |  |  |
| P value summary |  |  |  |  |  |
| Is there significant matching (P < 0.05)? | No |  |  |  |  |
|  |  |  |  |  |  |
| Data summary |  |  |  |  |  |
| Number of columns (Population) | 3 |  |  |  |  |
| Number of rows (Environment) | 4 |  |  |  |  |
| Number of subjects (Subject) | 21 |  |  |  |  |
| Number of missing values | 0 |  |  |  |  |
| Number of missing values | 0 |  |  |  |  |
|  |  |  |  |  |  |
| Within each row, compare columns (simple effects within rows) | |  |  |  |  |
|  |  |  |  |  |  |
| Number of families | 4 |  |  |  |  |
| Number of comparisons per family | 3 |  |  |  |  |
| Alpha | 0.05 |  |  |  |  |
|  |  |  |  |  |  |
| Bonferroni's multiple comparisons test | Mean Diff. | 95.00% CI of diff. | Significant? | Summary | Adjusted P Value |
|  |  |  |  |  |  |
| Stand diet |  |  |  |  |  |
| Population A vs. Population B | -0.491 | -0.6959 to -0.2861 | Yes | **** | <0.0001 |
| Population A vs. Population Exp | -0.3616 | -0.5665 to -0.1567 | Yes | *** | 0.0001 |
| Population B vs. Population Exp | 0.1294 | -0.07550 to 0.3343 | No | ns | 0.378 |
|  |  |  |  |  |  |
| Rest diet |  |  |  |  |  |
| Population A vs. Population B | -0.3069 | -0.5119 to -0.1020 | Yes | ** | 0.0014 |
| Population A vs. Population Exp | -0.4333 | -0.6382 to -0.2284 | Yes | **** | <0.0001 |
| Population B vs. Population Exp | -0.1263 | -0.3312 to 0.07858 | No | ns | 0.4053 |
|  |  |  |  |  |  |
| Infec diet |  |  |  |  |  |
| Population A vs. Population B | -0.1181 | -0.3230 to 0.08681 | No | ns | 0.4862 |
| Population A vs. Population Exp | -0.3172 | -0.5221 to -0.1123 | Yes | *** | 0.0009 |
| Population B vs. Population Exp | -0.1991 | -0.4040 to 0.005778 | No | ns | 0.0596 |
|  |  |  |  |  |  |
| Infec+Rest |  |  |  |  |  |
| Population A vs. Population B | -0.1527 | -0.3576 to 0.05216 | No | ns | 0.2154 |
| Population A vs. Population Exp | -0.1255 | -0.3304 to 0.07937 | No | ns | 0.4126 |
| Population B vs. Population Exp | 0.02721 | -0.1777 to 0.2321 | No | ns | >0.9999 |
|  |  |  |  |  |  |
| Within each column, compare rows (simple effects within columns) | |  |  |  |  |
|  |  |  |  |  |  |
| Number of families | 3 |  |  |  |  |
| Number of comparisons per family | 6 |  |  |  |  |
| Alpha | 0.05 |  |  |  |  |
|  |  |  |  |  |  |
| Bonferroni's multiple comparisons test | Mean Diff. | 95.00% CI of diff. | Significant? | Summary | Adjusted P Value |
|  |  |  |  |  |  |
| Population A |  |  |  |  |  |
| Stand diet vs. Rest diet | 0.0122 | -0.2146 to 0.2390 | No | ns | >0.9999 |
| Stand diet vs. Infec diet | 0.2056 | -0.02115 to 0.4324 | No | ns | 0.0978 |
| Stand diet vs. Infec+Rest | 0.2538 | 0.02700 to 0.4806 | Yes | * | 0.02 |
| Rest diet vs. Infec diet | 0.1934 | -0.03335 to 0.4202 | No | ns | 0.1411 |
| Rest diet vs. Infec+Rest | 0.2416 | 0.01480 to 0.4684 | Yes | * | 0.0305 |
| Infec diet vs. Infec+Rest | 0.04815 | -0.1786 to 0.2749 | No | ns | >0.9999 |
|  |  |  |  |  |  |
| Population B |  |  |  |  |  |
| Stand diet vs. Rest diet | 0.1963 | -0.03053 to 0.4231 | No | ns | 0.1298 |
| Stand diet vs. Infec diet | 0.5786 | 0.3518 to 0.8054 | Yes | **** | <0.0001 |
| Stand diet vs. Infec+Rest | 0.5921 | 0.3653 to 0.8189 | Yes | **** | <0.0001 |
| Rest diet vs. Infec diet | 0.3823 | 0.1555 to 0.6091 | Yes | *** | 0.0001 |
| Rest diet vs. Infec+Rest | 0.3958 | 0.1690 to 0.6226 | Yes | **** | <0.0001 |
| Infec diet vs. Infec+Rest | 0.0135 | -0.2133 to 0.2403 | No | ns | >0.9999 |
|  |  |  |  |  |  |
| Population Exp |  |  |  |  |  |
| Stand diet vs. Rest diet | -0.05946 | -0.2863 to 0.1673 | No | ns | >0.9999 |
| Stand diet vs. Infec diet | 0.25 | 0.02325 to 0.4768 | Yes | * | 0.0228 |
| Stand diet vs. Infec+Rest | 0.4899 | 0.2631 to 0.7167 | Yes | **** | <0.0001 |
| Rest diet vs. Infec diet | 0.3095 | 0.08271 to 0.5363 | Yes | ** | 0.0025 |
| Rest diet vs. Infec+Rest | 0.5493 | 0.3225 to 0.7761 | Yes | **** | <0.0001 |
| Infec diet vs. Infec+Rest | 0.2398 | 0.01303 to 0.4666 | Yes | * | 0.0324 |
|  |  |  |  |  |  |

Statistics of proteins

| Table Analyzed | Data protein |  |  |  |  |
| --- | --- | --- | --- | --- | --- |
|  |  |  |  |  |  |
| Mixed-effects model (REML) | Matching: Stacked | |  |  |  |
| Assume sphericity? | Yes |  |  |  |  |
| Alpha | 0.05 |  |  |  |  |
|  |  |  |  |  |  |
| Fixed effects (type III) | P value | P value summary | Statistically significant (P < 0.05)? | F (DFn, DFd) |  |
| Environment | 0.0818 | ns | No | F (3, 36) = 2.422 |  |
| Population | 0.0081 | ** | Yes | F (2, 12) = 7.395 |  |
| Environment x Population | <0.0001 | **** | Yes | F (6, 36) = 22.67 |  |
|  |  |  |  |  |  |
| Random effects | SD | Variance |  |  |  |
| Subject | 0.9584 | 0.9186 |  |  |  |
| Residual | 3.846 | 14.79 |  |  |  |
|  |  |  |  |  |  |
| Was the matching effective? |  |  |  |  |  |
| Chi-square, df | 0.2296, 1 |  |  |  |  |
| P value | 0.6318 |  |  |  |  |
| P value summary | ns |  |  |  |  |
| Is there significant matching (P < 0.05)? | No |  |  |  |  |
|  |  |  |  |  |  |
| Data summary |  |  |  |  |  |
| Number of columns (Population) | 3 |  |  |  |  |
| Number of rows (Environment) | 4 |  |  |  |  |
| Number of subjects (Subject) | 15 |  |  |  |  |
| Number of missing values | 0 |  |  |  |  |
|  |  |  |  |  |  |
| Within each row, compare columns (simple effects within rows) | | |  |  |  |
|  |  |  |  |  |  |
| Number of families | 4 |  |  |  |  |
| Number of comparisons per family | 3 |  |  |  |  |
| Alpha | 0.05 |  |  |  |  |
|  |  |  |  |  |  |
| Bonferroni's multiple comparisons test | Mean Diff. | 95.00% CI of diff. | Significant? | Summary | Adjusted P Value |
|  |  |  |  |  |  |
| Stand diet |  |  |  |  |  |
| Population A vs. Population B | -12.08 | -18.30 to -5.866 | Yes | **** | <0.0001 |
| Population A vs. Population Exp | 1.068 | -5.150 to 7.286 | No | ns | >0.9999 |
| Population B vs. Population Exp | 13.15 | 6.934 to 19.37 | Yes | **** | <0.0001 |
|  |  |  |  |  |  |
| Rest diet |  |  |  |  |  |
| Population A vs. Population B | -6.792 | -13.01 to -0.5737 | Yes | * | 0.0279 |
| Population A vs. Population Exp | -7.5 | -13.72 to -1.282 | Yes | * | 0.0131 |
| Population B vs. Population Exp | -0.708 | -6.926 to 5.510 | No | ns | >0.9999 |
|  |  |  |  |  |  |
| Infec diet |  |  |  |  |  |
| Population A vs. Population B | 16.94 | 10.73 to 23.16 | Yes | **** | <0.0001 |
| Population A vs. Population Exp | 13.78 | 7.558 to 19.99 | Yes | **** | <0.0001 |
| Population B vs. Population Exp | -3.168 | -9.386 to 3.050 | No | ns | 0.6371 |
|  |  |  |  |  |  |
| Infec+Rest |  |  |  |  |  |
| Population A vs. Population B | 16.76 | 10.55 to 22.98 | Yes | **** | <0.0001 |
| Population A vs. Population Exp | 12.83 | 6.610 to 19.05 | Yes | **** | <0.0001 |
| Population B vs. Population Exp | -3.936 | -10.15 to 2.282 | No | ns | 0.3688 |
|  |  |  |  |  |  |
|  |  |  |  |  |  |
| Within each column, compare rows (simple effects within columns) | | |  |  |  |
|  |  |  |  |  |  |
| Number of families | 3 |  |  |  |  |
| Number of comparisons per family | 6 |  |  |  |  |
| Alpha | 0.05 |  |  |  |  |
|  |  |  |  |  |  |
| Bonferroni's multiple comparisons test | Mean Diff. | 95.00% CI of diff. | Significant? | Summary | Adjusted P Value |
|  |  |  |  |  |  |
| Population A |  |  |  |  |  |
| Stand diet vs. Rest diet | 0.612 | -6.179 to 7.403 | No | ns | >0.9999 |
| Stand diet vs. Infec diet | -16.24 | -23.03 to -9.445 | Yes | **** | <0.0001 |
| Stand diet vs. Infec+Rest | -16.82 | -23.61 to -10.03 | Yes | **** | <0.0001 |
| Rest diet vs. Infec diet | -16.85 | -23.64 to -10.06 | Yes | **** | <0.0001 |
| Rest diet vs. Infec+Rest | -17.44 | -24.23 to -10.65 | Yes | **** | <0.0001 |
| Infec diet vs. Infec+Rest | -0.588 | -7.379 to 6.203 | No | ns | >0.9999 |
|  |  |  |  |  |  |
| Population B |  |  |  |  |  |
| Stand diet vs. Rest diet | 5.904 | -0.8866 to 12.69 | No | ns | 0.122 |
| Stand diet vs. Infec diet | 12.79 | 6.001 to 19.58 | Yes | **** | <0.0001 |
| Stand diet vs. Infec+Rest | 12.02 | 5.233 to 18.81 | Yes | *** | 0.0001 |
| Rest diet vs. Infec diet | 6.888 | 0.09738 to 13.68 | Yes | * | 0.0452 |
| Rest diet vs. Infec+Rest | 6.12 | -0.6706 to 12.91 | No | ns | 0.0988 |
| Infec diet vs. Infec+Rest | -0.768 | -7.559 to 6.023 | No | ns | >0.9999 |
|  |  |  |  |  |  |
| Population Exp |  |  |  |  |  |
| Stand diet vs. Rest diet | -7.956 | -14.75 to -1.165 | Yes | * | 0.0142 |
| Stand diet vs. Infec diet | -3.528 | -10.32 to 3.263 | No | ns | 0.9334 |
| Stand diet vs. Infec+Rest | -5.064 | -11.85 to 1.727 | No | ns | 0.267 |
| Rest diet vs. Infec diet | 4.428 | -2.363 to 11.22 | No | ns | 0.462 |
| Rest diet vs. Infec+Rest | 2.892 | -3.899 to 9.683 | No | ns | >0.9999 |
| Infec diet vs. Infec+Rest | -1.536 | -8.327 to 5.255 | No | ns | >0.9999 |

Statistics of lipids

| Table Analyzed | Data lipid |  |  |  |  |
| --- | --- | --- | --- | --- | --- |
|  |  |  |  |  |  |
| Mixed-effects model (REML) | Matching: Stacked |  |  |  |  |
| Assume sphericity? | Yes |  |  |  |  |
| Alpha | 0.05 |  |  |  |  |
|  |  |  |  |  |  |
| Fixed effects (type III) | P value | P value summary | Statistically significant (P < 0.05)? | F (DFn, DFd) |  |
| Environment | <0.0001 | **** | Yes | F (3, 36) = 12.75 |  |
| Population | <0.0001 | **** | Yes | F (2, 12) = 24.74 |  |
| Environment x Population | <0.0001 | **** | Yes | F (6, 36) = 13.77 |  |
|  |  |  |  |  |  |
| Random effects | SD | Variance |  |  |  |
| Subject | 0.06479 | 0.004198 |  |  |  |
| Residual | 0.4082 | 0.1666 |  |  |  |
|  |  |  |  |  |  |
| Was the matching effective? | |  |  |  |  |
| Chi-square, df | 0.04215, 1 |  |  |  |  |
| P value | 0.8373 |  |  |  |  |
| P value summary | ns |  |  |  |  |
| Is there significant matching (P < 0.05)? | No |  |  |  |  |
|  |  |  |  |  |  |
| Data summary |  |  |  |  |  |
| Number of columns (Population) | 3 |  |  |  |  |
| Number of rows (Environment) | 4 |  |  |  |  |
| Number of subjects (Subject) | 15 |  |  |  |  |
| Number of missing values | 0 |  |  |  |  |
|  |  |  |  |  |  |
| Within each row, compare columns (simple effects within rows) | | |  |  |  |
|  |  |  |  |  |  |
| Number of families | 4 |  |  |  |  |
| Number of comparisons per family | 3 |  |  |  |  |
| Alpha | 0.05 |  |  |  |  |
|  |  |  |  |  |  |
| Bonferroni's multiple comparisons test | Mean Diff. | 95.00% CI of diff. | Significant? | Summary | Adjusted P Value |
|  |  |  |  |  |  |
| Stand diet |  |  |  |  |  |
| Population A vs. Population B | 0.612 | -0.03650 to 1.260 | No | ns | 0.0703 |
| Population A vs. Population Exp | -0.46 | -1.108 to 0.1885 | No | ns | 0.2545 |
| Population B vs. Population Exp | -1.072 | -1.720 to -0.4235 | Yes | *** | 0.0005 |
|  |  |  |  |  |  |
| Rest diet |  |  |  |  |  |
| Population A vs. Population B | -1.776 | -2.424 to -1.128 | Yes | **** | <0.0001 |
| Population A vs. Population Exp | -0.882 | -1.530 to -0.2335 | Yes | ** | 0.0044 |
| Population B vs. Population Exp | 0.894 | 0.2455 to 1.542 | Yes | ** | 0.0039 |
|  |  |  |  |  |  |
| Infec diet |  |  |  |  |  |
| Population A vs. Population B | 0.618 | -0.03050 to 1.266 | No | ns | 0.0665 |
| Population A vs. Population Exp | -0.614 | -1.262 to 0.03450 | No | ns | 0.069 |
| Population B vs. Population Exp | -1.232 | -1.880 to -0.5835 | Yes | **** | <0.0001 |
|  |  |  |  |  |  |
| Infec+Rest |  |  |  |  |  |
| Population A vs. Population B | -0.068 | -0.7165 to 0.5805 | No | ns | >0.9999 |
| Population A vs. Population Exp | -1.608 | -2.256 to -0.9595 | Yes | **** | <0.0001 |
| Population B vs. Population Exp | -1.54 | -2.188 to -0.8915 | Yes | **** | <0.0001 |
|  |  |  |  |  |  |
| Within each column, compare rows (simple effects within columns) | | |  |  |  |
|  |  |  |  |  |  |
| Number of families | 3 |  |  |  |  |
| Number of comparisons per family | 6 |  |  |  |  |
| Alpha | 0.05 |  |  |  |  |
|  |  |  |  |  |  |
| Bonferroni's multiple comparisons test | Mean Diff. | 95.00% CI of diff. | Significant? | Summary | Adjusted P Value |
|  |  |  |  |  |  |
| Population A |  |  |  |  |  |
| Stand diet vs. Rest diet | 0.194 | -0.5268 to 0.9148 | No | ns | >0.9999 |
| Stand diet vs. Infec diet | -0.002 | -0.7228 to 0.7188 | No | ns | >0.9999 |
| Stand diet vs. Infec+Rest | 0.016 | -0.7048 to 0.7368 | No | ns | >0.9999 |
| Rest diet vs. Infec diet | -0.196 | -0.9168 to 0.5248 | No | ns | >0.9999 |
| Rest diet vs. Infec+Rest | -0.178 | -0.8988 to 0.5428 | No | ns | >0.9999 |
| Infec diet vs. Infec+Rest | 0.018 | -0.7028 to 0.7388 | No | ns | >0.9999 |
|  |  |  |  |  |  |
| Population B |  |  |  |  |  |
| Stand diet vs. Rest diet | -2.194 | -2.915 to -1.473 | Yes | **** | <0.0001 |
| Stand diet vs. Infec diet | 0.004 | -0.7168 to 0.7248 | No | ns | >0.9999 |
| Stand diet vs. Infec+Rest | -0.664 | -1.385 to 0.05682 | No | ns | 0.0863 |
| Rest diet vs. Infec diet | 2.198 | 1.477 to 2.919 | Yes | **** | <0.0001 |
| Rest diet vs. Infec+Rest | 1.53 | 0.8092 to 2.251 | Yes | **** | <0.0001 |
| Infec diet vs. Infec+Rest | -0.668 | -1.389 to 0.05282 | No | ns | 0.0831 |
|  |  |  |  |  |  |
| Population Exp |  |  |  |  |  |
| Stand diet vs. Rest diet | -0.228 | -0.9488 to 0.4928 | No | ns | >0.9999 |
| Stand diet vs. Infec diet | -0.156 | -0.8768 to 0.5648 | No | ns | >0.9999 |
| Stand diet vs. Infec+Rest | -1.132 | -1.853 to -0.4112 | Yes | *** | 0.0006 |
| Rest diet vs. Infec diet | 0.072 | -0.6488 to 0.7928 | No | ns | >0.9999 |
| Rest diet vs. Infec+Rest | -0.904 | -1.625 to -0.1832 | Yes | ** | 0.0075 |
| Infec diet vs. Infec+Rest | -0.976 | -1.697 to -0.2552 | Yes | ** | 0.0034 |

Statistics of Phenol oxidase

| Table Analyzed | Data PO |  |  |  |  |
| --- | --- | --- | --- | --- | --- |
|  |  |  |  |  |  |
| Mixed-effects model (REML) | Matching: Stacked | |  |  |  |
| Assume sphericity? | Yes |  |  |  |  |
| Alpha | 0.05 |  |  |  |  |
|  |  |  |  |  |  |
| Fixed effects (type III) | P value | P value summary | Statistically significant (P < 0.05)? | F (DFn, DFd) |  |
| Environment | <0.0001 | **** | Yes | F (3, 36) = 29.58 |  |
| Population | 0.3337 | ns | No | F (2, 12) = 1.204 |  |
| Environment x Population | <0.0001 | **** | Yes | F (6, 36) = 11.14 |  |
|  |  |  |  |  |  |
| Random effects | SD | Variance |  |  |  |
| Subject | 2.383 | 5.681 |  |  |  |
| Residual | 7.014 | 49.19 |  |  |  |
|  |  |  |  |  |  |
| Was the matching effective? | |  |  |  |  |
| Chi-square, df | 0.6887, 1 |  |  |  |  |
| P value | 0.4066 |  |  |  |  |
| P value summary | ns |  |  |  |  |
| Is there significant matching (P < 0.05)? | No |  |  |  |  |
|  |  |  |  |  |  |
| Data summary |  |  |  |  |  |
| Number of columns (Population) | 3 |  |  |  |  |
| Number of rows (Environment) | 4 |  |  |  |  |
| Number of subjects (Subject) | 15 |  |  |  |  |
| Number of missing values | 0 |  |  |  |  |
|  |  |  |  |  |  |
| Within each row, compare columns (simple effects within rows) | | | |  |  |
|  |  |  |  |  |  |
| Number of families | 4 |  |  |  |  |
| Number of comparisons per family | 3 |  |  |  |  |
| Alpha | 0.05 |  |  |  |  |
|  |  |  |  |  |  |
| Bonferroni's multiple comparisons test | Mean Diff. | 95.00% CI of diff. | Significant? | Summary | Adjusted P Value |
|  |  |  |  |  |  |
| Stand diet |  |  |  |  |  |
| Population A vs. Population B | 31.2 | 19.58 to 42.83 | Yes | **** | <0.0001 |
| Population A vs. Population Exp | 9.069 | -2.554 to 20.69 | No | ns | 0.1764 |
| Population B vs. Population Exp | -22.13 | -33.76 to -10.51 | Yes | **** | <0.0001 |
|  |  |  |  |  |  |
| Rest diet |  |  |  |  |  |
| Population A vs. Population B | 2.098 | -9.525 to 13.72 | No | ns | >0.9999 |
| Population A vs. Population Exp | 2.821 | -8.802 to 14.44 | No | ns | >0.9999 |
| Population B vs. Population Exp | 0.7227 | -10.90 to 12.35 | No | ns | >0.9999 |
|  |  |  |  |  |  |
| Infec diet |  |  |  |  |  |
| Population A vs. Population B | -6.994 | -18.62 to 4.629 | No | ns | 0.4261 |
| Population A vs. Population Exp | -12.23 | -23.86 to -0.6120 | Yes | * | 0.036 |
| Population B vs. Population Exp | -5.241 | -16.86 to 6.382 | No | ns | 0.8065 |
|  |  |  |  |  |  |
| Infec+Rest |  |  |  |  |  |
| Population A vs. Population B | -12.4 | -24.02 to -0.7760 | Yes | * | 0.0329 |
| Population A vs. Population Exp | -0.6239 | -12.25 to 11.00 | No | ns | >0.9999 |
| Population B vs. Population Exp | 11.77 | 0.1521 to 23.40 | Yes | * | 0.0461 |
|  |  |  |  |  |  |
| Within each column, compare rows (simple effects within columns) | | | |  |  |
|  |  |  |  |  |  |
| Number of families | 3 |  |  |  |  |
| Number of comparisons per family | 6 |  |  |  |  |
| Alpha | 0.05 |  |  |  |  |
|  |  |  |  |  |  |
| Bonferroni's multiple comparisons test | Mean Diff. | 95.00% CI of diff. | Significant? | Summary | Adjusted P Value |
|  |  |  |  |  |  |
| Population A |  |  |  |  |  |
| Stand diet vs. Rest diet | 29.41 | 17.03 to 41.80 | Yes | **** | <0.0001 |
| Stand diet vs. Infec diet | 27.88 | 15.49 to 40.26 | Yes | **** | <0.0001 |
| Stand diet vs. Infec+Rest | 39.75 | 27.37 to 52.14 | Yes | **** | <0.0001 |
| Rest diet vs. Infec diet | -1.536 | -13.92 to 10.85 | No | ns | >0.9999 |
| Rest diet vs. Infec+Rest | 10.34 | -2.049 to 22.72 | No | ns | 0.1532 |
| Infec diet vs. Infec+Rest | 11.87 | -0.5130 to 24.26 | No | ns | 0.0668 |
|  |  |  |  |  |  |
| Population B |  |  |  |  |  |
| Stand diet vs. Rest diet | 0.3095 | -12.08 to 12.69 | No | ns | >0.9999 |
| Stand diet vs. Infec diet | -10.32 | -22.70 to 2.067 | No | ns | 0.1546 |
| Stand diet vs. Infec+Rest | -3.851 | -16.24 to 8.534 | No | ns | >0.9999 |
| Rest diet vs. Infec diet | -10.63 | -23.01 to 1.757 | No | ns | 0.1314 |
| Rest diet vs. Infec+Rest | -4.161 | -16.55 to 8.224 | No | ns | >0.9999 |
| Infec diet vs. Infec+Rest | 6.467 | -5.918 to 18.85 | No | ns | 0.9213 |
|  |  |  |  |  |  |
| Population Exp |  |  |  |  |  |
| Stand diet vs. Rest diet | 23.17 | 10.78 to 35.55 | Yes | **** | <0.0001 |
| Stand diet vs. Infec diet | 6.575 | -5.810 to 18.96 | No | ns | 0.8818 |
| Stand diet vs. Infec+Rest | 30.06 | 17.67 to 42.44 | Yes | **** | <0.0001 |
| Rest diet vs. Infec diet | -16.59 | -28.98 to -4.206 | Yes | ** | 0.0038 |
| Rest diet vs. Infec+Rest | 6.891 | -5.494 to 19.28 | No | ns | 0.7743 |
| Infec diet vs. Infec+Rest | 23.48 | 11.10 to 35.87 | Yes | **** | <0.0001 |

Statistics of antibacterial

| Table Analyzed | Transform of Transform of Data antibacterial | |  |  |  |
| --- | --- | --- | --- | --- | --- |
|  |  |  |  |  |  |
| Mixed-effects model (REML) | Matching: Stacked |  |  |  |  |
| Assume sphericity? | Yes |  |  |  |  |
| Alpha | 0.05 |  |  |  |  |
|  |  |  |  |  |  |
| Fixed effects (type III) | P value | P value summary | Statistically significant (P < 0.05)? | F (DFn, DFd) |  |
| Environment | <0.0001 | **** | Yes | F (3, 36) = 551.4 |  |
| Population | <0.0001 | **** | Yes | F (2, 12) = 672.7 |  |
| Environment x Population | <0.0001 | **** | Yes | F (6, 36) = 13.67 |  |
|  |  |  |  |  |  |
| Random effects | SD | Variance |  |  |  |
| Subject | 0.005798 | 0.00003361 |  |  |  |
| Residual | 0.02068 | 0.0004275 |  |  |  |
|  |  |  |  |  |  |
| Was the matching effective? |  |  |  |  |  |
| Chi-square, df | 0.3515, 1 |  |  |  |  |
| P value | 0.5532 |  |  |  |  |
| P value summary | ns |  |  |  |  |
| Is there significant matching (P < 0.05)? | No |  |  |  |  |
|  |  |  |  |  |  |
| Data summary |  |  |  |  |  |
| Number of columns (Population) | 3 |  |  |  |  |
| Number of rows (Environment) | 4 |  |  |  |  |
| Number of subjects (Subject) | 15 |  |  |  |  |
| Number of missing values | 0 |  |  |  |  |
|  |  |  |  |  |  |
| Within each row, compare columns (simple effects within rows) | |  |  |  |  |
|  |  |  |  |  |  |
| Number of families | 4 |  |  |  |  |
| Number of comparisons per family | 3 |  |  |  |  |
| Alpha | 0.05 |  |  |  |  |
|  |  |  |  |  |  |
| Bonferroni's multiple comparisons test | Mean Diff. | 95.00% CI of diff. | Significant? | Summary | Adjusted P Value |
|  |  |  |  |  |  |
| Stand diet |  |  |  |  |  |
| Population A vs. Population B | 0.2164 | 0.1827 to 0.2501 | Yes | **** | <0.0001 |
| Population A vs. Population Exp | 0.2123 | 0.1786 to 0.2460 | Yes | **** | <0.0001 |
| Population B vs. Population Exp | -0.004131 | -0.03782 to 0.02956 | No | ns | >0.9999 |
|  |  |  |  |  |  |
| Rest diet |  |  |  |  |  |
| Population A vs. Population B | 0.2508 | 0.2172 to 0.2845 | Yes | **** | <0.0001 |
| Population A vs. Population Exp | 0.1874 | 0.1537 to 0.2211 | Yes | **** | <0.0001 |
| Population B vs. Population Exp | -0.06347 | -0.09716 to -0.02978 | Yes | **** | <0.0001 |
|  |  |  |  |  |  |
| Infec diet |  |  |  |  |  |
| Population A vs. Population B | 0.3381 | 0.3044 to 0.3718 | Yes | **** | <0.0001 |
| Population A vs. Population Exp | 0.2955 | 0.2618 to 0.3292 | Yes | **** | <0.0001 |
| Population B vs. Population Exp | -0.04254 | -0.07623 to -0.008845 | Yes | ** | 0.0089 |
|  |  |  |  |  |  |
| Infec+Rest |  |  |  |  |  |
| Population A vs. Population B | 0.2147 | 0.1810 to 0.2484 | Yes | **** | <0.0001 |
| Population A vs. Population Exp | 0.1712 | 0.1375 to 0.2049 | Yes | **** | <0.0001 |
| Population B vs. Population Exp | -0.04349 | -0.07718 to -0.009800 | Yes | ** | 0.0073 |
|  |  |  |  |  |  |
| Within each column, compare rows (simple effects within columns) | | |  |  |  |
|  |  |  |  |  |  |
| Number of families | 3 |  |  |  |  |
| Number of comparisons per family | 6 |  |  |  |  |
| Alpha | 0.05 |  |  |  |  |
|  |  |  |  |  |  |
| Bonferroni's multiple comparisons test | Mean Diff. | 95.00% CI of diff. | Significant? | Summary | Adjusted P Value |
|  |  |  |  |  |  |
| Population A |  |  |  |  |  |
| Stand diet vs. Rest diet | 0.2454 | 0.2089 to 0.2819 | Yes | **** | <0.0001 |
| Stand diet vs. Infec diet | 0.1191 | 0.08263 to 0.1556 | Yes | **** | <0.0001 |
| Stand diet vs. Infec+Rest | 0.2942 | 0.2577 to 0.3307 | Yes | **** | <0.0001 |
| Rest diet vs. Infec diet | -0.1263 | -0.1628 to -0.08977 | Yes | **** | <0.0001 |
| Rest diet vs. Infec+Rest | 0.0488 | 0.01229 to 0.08531 | Yes | ** | 0.0039 |
| Infec diet vs. Infec+Rest | 0.1751 | 0.1386 to 0.2116 | Yes | **** | <0.0001 |
|  |  |  |  |  |  |
| Population B |  |  |  |  |  |
| Stand diet vs. Rest diet | 0.2798 | 0.2433 to 0.3164 | Yes | **** | <0.0001 |
| Stand diet vs. Infec diet | 0.2408 | 0.2043 to 0.2773 | Yes | **** | <0.0001 |
| Stand diet vs. Infec+Rest | 0.2925 | 0.2560 to 0.3290 | Yes | **** | <0.0001 |
| Rest diet vs. Infec diet | -0.03906 | -0.07557 to -0.002550 | Yes | * | 0.0303 |
| Rest diet vs. Infec+Rest | 0.01263 | -0.02388 to 0.04914 | No | ns | >0.9999 |
| Infec diet vs. Infec+Rest | 0.05169 | 0.01518 to 0.08820 | Yes | ** | 0.0021 |
|  |  |  |  |  |  |
| Population Exp |  |  |  |  |  |
| Stand diet vs. Rest diet | 0.2205 | 0.1840 to 0.2570 | Yes | **** | <0.0001 |
| Stand diet vs. Infec diet | 0.2024 | 0.1659 to 0.2389 | Yes | **** | <0.0001 |
| Stand diet vs. Infec+Rest | 0.2531 | 0.2166 to 0.2896 | Yes | **** | <0.0001 |
| Rest diet vs. Infec diet | -0.01813 | -0.05463 to 0.01838 | No | ns | >0.9999 |
| Rest diet vs. Infec+Rest | 0.0326 | -0.003904 to 0.06911 | No | ns | 0.1043 |
| Infec diet vs. Infec+Rest | 0.05073 | 0.01422 to 0.08724 | Yes | ** | 0.0026 |
